# The promise of AlphaFold for gene structure annotation

**DOI:** 10.1093/nar/gkag369

**Published:** 2026-04-24

**Authors:** Helen R Davison, Ulrike Böhme, Shahram Mesdaghi, Paul A Wilkinson, David S Roos, Andrew R Jones, Daniel J Rigden

**Affiliations:** Department of Biochemistry, Cell and Systems Biology, Institute of Systems, Molecular and Integrative Biology, University of Liverpool, Liverpool, L69 7ZB, United Kingdom; Department of Biochemistry, Cell and Systems Biology, Institute of Systems, Molecular and Integrative Biology, University of Liverpool, Liverpool, L69 7ZB, United Kingdom; Department of Biochemistry, Cell and Systems Biology, Institute of Systems, Molecular and Integrative Biology, University of Liverpool, Liverpool, L69 7ZB, United Kingdom; Computational Biology Facility, University of Liverpool, Liverpool, L69 7ZB, United Kingdom; Department of Biochemistry, Cell and Systems Biology, Institute of Systems, Molecular and Integrative Biology, University of Liverpool, Liverpool, L69 7ZB, United Kingdom; Department of Biology, University of Pennsylvania, Philadelphia, PA 19104, United States; Department of Biochemistry, Cell and Systems Biology, Institute of Systems, Molecular and Integrative Biology, University of Liverpool, Liverpool, L69 7ZB, United Kingdom; Department of Biochemistry, Cell and Systems Biology, Institute of Systems, Molecular and Integrative Biology, University of Liverpool, Liverpool, L69 7ZB, United Kingdom

## Abstract

Most new genomes lack annotation, automated methods are error-prone, and few genomes are ever manually curated due to time and cost. Protein structure predictions may offer a new route to assess and improve gene models without requiring experimental data. Here, we explore whether scores from protein structure prediction can aid in scoring gene model quality. We chose three species (*Fusarium graminearum, Toxoplasma gondii*, and *Aspergillus fumigatus*) from the VEuPathDB database that have collectively undergone more than 1000 manual curation events. We modelled translations of the gene models with AlphaFold 3, before and after curation, collecting various scores. Then we carried out structure searching of the PDB with Foldseek and sequence-based domain identification using InterProScan. We profiled the scores produced by these methods to identify those best for gene model assessment. AlphaFold 3 scores strongly favoured manually improved over pre-improvement gene models, supporting 65–84% of manually-curated changes. Combining scores across multiple tools (AlphaFold 3, Foldseek and InterProScan) provided further improvements in model scoring. Overall, the most discriminative scores combined the outputs of AlphaFold 3 and Foldseek. Importantly, we find that scores from the much faster Protenix-Mini retain the same discriminatory power as those from AlphaFold 3. Our results, therefore, highlight the potential of scores derived from deep learning-based protein structure prediction for scoring gene models in the absence of experimental data.

## Graphical Abstract

**Graphical Abstract:**
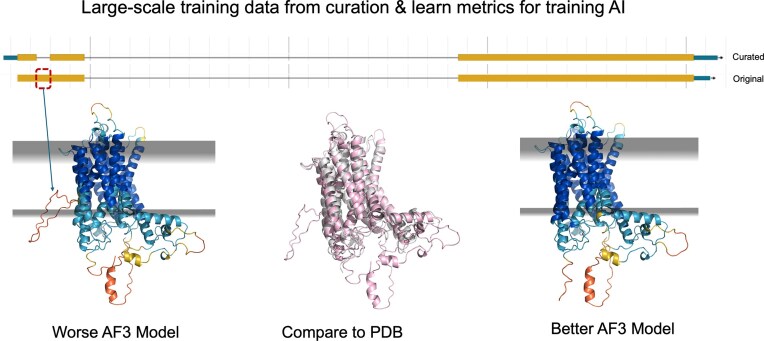

## Introduction

The growing accessibility of genome sequencing means that genome assemblies are increasingly available for many organisms and isolates, yet gene structure annotation remains a challenge. Since the beginning of genome sequencing efforts, extensive manual curation of model organisms has corrected exon-intron structure and start codons genome-wide [1]. However, the efficacy of manual curation is dependent on the availability of large amounts of high-quality experimental data and highly trained and specialised curators. Many of the same challenges for accurate next-generation gene annotation persist after decades of work [2]. Newly sequenced genomes for less well-studied organisms rarely have the depth of experimental data, nor funding for curators to take a gene-by-gene approach to improvements [3]. Thus, alternative approaches will be required for the increasing number of species whose genomes have been sequenced but for which functional genomics datasets are unavailable [4, 5].

Genome-wide, automated eukaryotic gene finding software were first developed in the mid 1990s, including GeneMark [6, 7], GENSCAN [8] and later AUGUSTUS [9], using intrinsic genomic signals for the identification of coding regions These packages were later incorporated into - or superseded by - methods using signals from protein sequences (e.g. orthologs), or experimental data (initially ESTs, and later RNASeq *e.g*. MAKER [10], BRAKER1/2/3 [10, 11], Gnomon/NCBI (https://www.ncbi.nlm.nih.gov/refseq/annotation_euk/process), Ensembl pipelines [12]) and more recently, deep learning *ab initio* pipelines such as Helixer [13]. All pipeline methods that incorporate multiple types of evidence have some internal methods for ranking plausible transcript models. For example, BRAKER2/3 uses TSEBRA to combine evidence from protein sequence orthology and RNASeq, thereby achieving better performance than with a single evidence type [11, 14]. BRAKER3 is considered state-of-the-art, capable of reaching F1 scores of 50–80% at a whole-gene level, but still yields a high number of predictions that may not reflect biological reality [11] (https://github.com/galaxyproject/training-material). A few pipelines produce user-accessible scores that can be used to evaluate the trustworthiness of gene models. MAKER2 and FINDER provide a per-model quality score called Average Edit Distance (AED) [15, 16]. AED is determined by the distance between a given gene model and the assembled transcript evidence from RNASeq. Other methods have been developed to allow gene model scoring independent of gene finding software. For example, TRaCE uses AED, InterPro domain coverage, and protein length, applied in a simple voting system [17]. PSAURON uses a neural network trained on NCBI RefSeq genomes to score the plausibility of gene models [18].

Here, building on previous ideas [19], we test to what extent the latest generation of deep learning-based protein structure prediction methods can provide fresh and complementary sources of information to support gene model prediction. The difficulties inherent in accurately identifying exons can result in missing or supernumerary exons, which impact the integrity and plausibility of the 3D protein structure in ways that should be detectable in protein models. Programs like AlphaFold 3 have revolutionised protein structural bioinformatics by enabling fast, accurate modelling of most proteins [20–22]. These tools, and other similar methods, provide reliable estimates of confidence in structural predictions, which is key to their utility and broad adoption.

**Figure 1.**
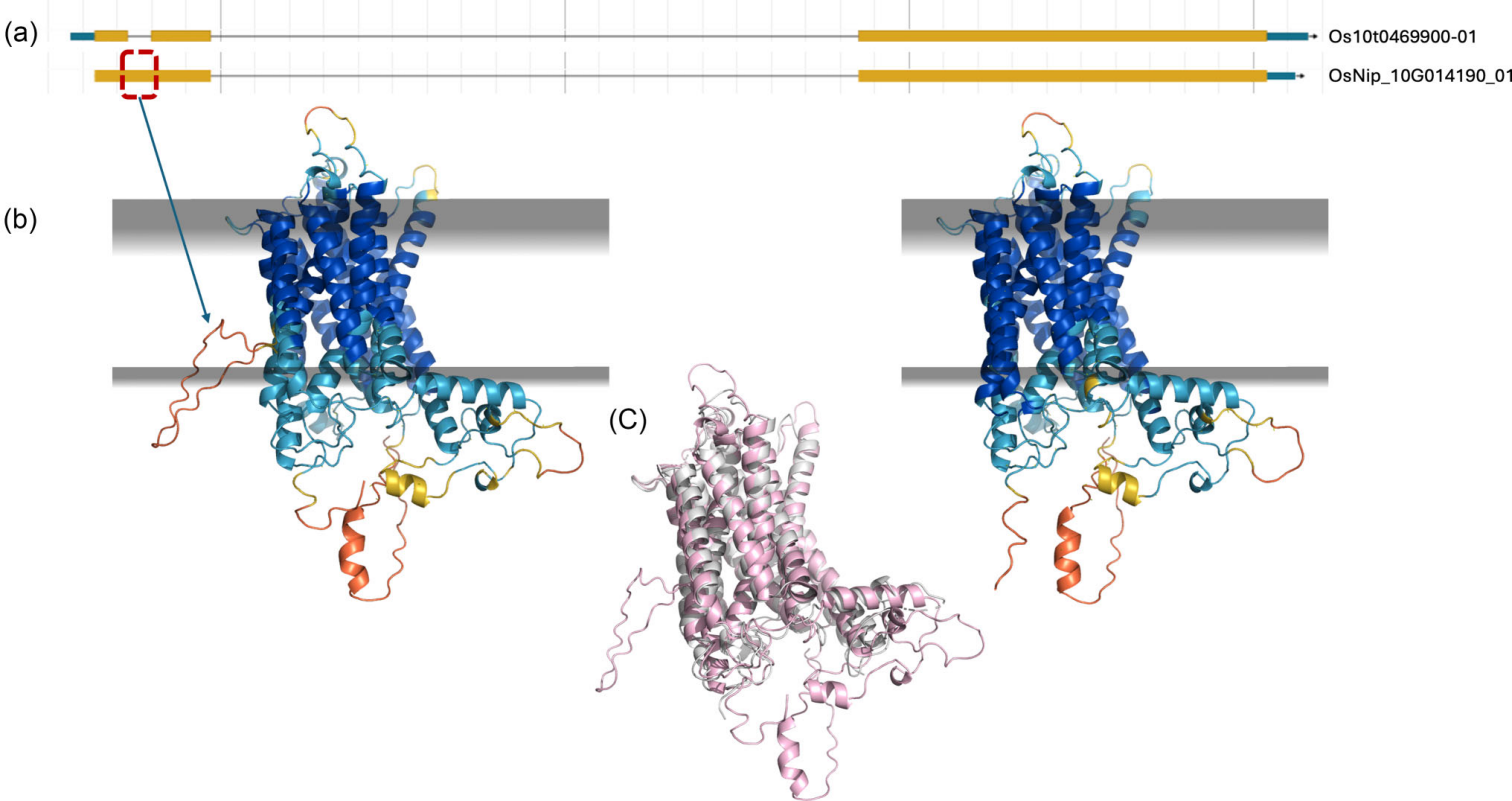
An illustrated example of how structure helps assess gene model quality. (**a**) Two gene models for Asian rice (*Oryza sativa*) TGF-beta receptor, type I/II extracellular region family protein from different annotation sources. OsNip_10G014190_01 (new gene model generated with long-read RNASeq from Gramene.org) predicts two exons, Os10t0469900-01 (RAP-DB) predicts three exons. (**b**) AlphaFold2 (ColabFold) structural models of OsNip_10G014190_01 and Os10t0469900-01. The models highlight a putative intron read-through that generates a low-confidence loop inconsistent with the tightly bundled α-helical structure. Grey planes represent predicted membrane boundaries calculated by the PPM server [25]. Notably, the introduced loop is positioned within the membrane, providing a clear indication of misannotation. Model confidence is indicated by pLDDT colouring, ranging from blue (high confidence) to orange (low confidence). Importantly, sequentially identical neighbouring residues of the introduced loop drop in confidence (cyan left, blue right) despite the Multiple Sequence Alignments generated during modelling of each having similar depth (Supplementary Fig. S1). (**c**) Structural alignment of the OsNip_10G014190_01 model (pink) with its closest structural neighbour identified by Foldseek, PDB entry 4oh3 (grey). The model extends through an intron, generating an erroneous loop absent from the structural database hit.

AlphaFold 3, which is our focus in this paper, produces three main scores: predicted local distance difference test (pLDDT), Predicted Aligned Error (PAE), and predicted Template Modelling score (pTM). The pLDDT provides a per-residue estimate of the probability that the local environment of a given amino acid in the model resembles that of the same position in the true structure. Regions with low pLDDT scores can result not only from unreliable prediction, but can also authentically reflect intrinsically disordered regions of the target protein that lack stable structure, at least in the absence of binding partners [23]. The PAE provides confidence in residue-residue separations for the whole structure, estimating the likely error on all residue pairs. And finally, the pTM is intended to provide an overall score for fold similarity (on a scale from 0 to 1, where 1 means identical structure) between the model and the true structure. One important note is that the pTM is depressed by the presence of low-confidence (e.g. intrinsic disorder) regions in the model [24]. Our preliminary results using AlphaFold 3 predictions from alternative gene annotations in the Asian rice (*Oryza sativa*) genome suggested that this approach is worth exploring (Fig. 1 shows an example). They demonstrate that subtle incorrect splicing signals can be detected in AlphaFold structures.

We do not expect that scores from AlphaFold 3 alone will always unambiguously identify the correct version of a gene amongst alternatives. Where a structure prediction strongly resembles an experimentally characterised protein it can be considered more likely to result from a correct gene model. Therefore, we further propose screening AlphaFold 3 results against databases such as the Protein Data Bank with Foldseek or InterPro with InterProScan [26–29] (Fig. 1).

In this paper, we test this novel application of AlphaFold 3 against the results of recent manual gene model curation exercises in three organisms (*Fusarium graminearum* str. PH-1, *Toxoplasma gondii* str. ME49, and *Aspergillus fumigatus* str. Af293). The organisms are held in the VEuPathDB database [30], and each can be considered a challenge for gene annotation. *Toxoplasma*, while well studied and annotated, is known for its highly disordered proteins, typical of *Apicomplexa* [31]. Fungi in general have hugely varying and complex genome structures, and tend to have higher gene density and fewer introns compared to other eukaryotes [32]. All three species were chosen because they have undergone substantial manual curation and have metadata records detailing the rationale in each case. We compare scores from AlphaFold 3, Foldseek, and InterProScan and find that each has excellent discriminatory power across all three organisms. Importantly, we find that there is complementarity between the scores, indicating that their combination with existing metrics in a machine learning framework could be useful for improving the performance of the next generation of gene finding tools.

## Materials and methods

### Selection of genomes and gene model pairs

Gene model annotations for *Toxoplasma gondii* str. ME49 underwent substantial changes on ToxoDB (A VEuPathDB sub-site; [33]) between build versions 65 and 66. Over 1200 genes were manually curated to improve or correct their structure. Metadata for altered gene models were retrieved from https://toxodb.org/toxo/app/static-content/ToxoDB/news.html#ToxoDB66Released. *Fusarium graminearum* and *Aspergillus fumigatus* underwent similar curation of 2047 and 1969 gene models, respectively, between build version 68 and a future release of their genomes on FungiDB [34, 35]. The records of these changes are currently publicly available on the Apollo community annotations track and can be viewed through JBrowse.

Methods for carrying out annotation can be found in the supplementary information. Files for all of the genomes used in this paper, including protein sequence files, gffs, and metadata, have been included as supplementary information. All data were retrieved from ToxoDB and FungiDB data download pages for relevant build version numbers (65 and 66 for *Toxoplasma gondii*, and 68 and internal data for the two fungi). All updates to gene models were carried out by Böhme through Apollo [36] using transcriptomics data and community annotations publicly available on VEuPathDB.

Once downloaded, protein sequences for new and old annotations were aligned and scored with BioPython version 1.85 [37]. Sequences were filtered for pairs that shared < 80% amino acid sequence identity and underwent edits classed as ‘changed’ (where boundaries of exons, introns, or UTRs had been amended). This resulted in 444, 317, and 461 gene pairs, respectively, from *T. gondii* str. ME49*, F. graminearum* str. PH-1 and *A. fumigatus* str. Af293 for new and old versions.

### Protein-based analyses

The sets of gene pairs with <80% sequence identity for each of the three organisms were run through structural prediction software AlphaFold 3 (AF3) [20]. Top-scoring results from AlphaFold 3 were then taken forward and run through Foldseek Release 10 [29] to find structural neighbours in the Protein Data Bank [38] as at January 2025. Both AlphaFold 3 and Foldseek were run on a HPC cluster system on GPU and CPU, respectively, through singularity containers. Per residue and per protein pLDDT scores were extracted and calculated from the AlphaFold 3 model output in Python. The AF3 modelling took around 126 h of GPU node time (corresponding largely to GPU use). For comparison of timings, modelling was also done using Protenix-Mini [39], a fast variant of the AF3-like model Protenix [40].

InterProScan version 5.73–104.0 [27] was run on the amino acid coding sequence with all applications, and the flags: –iprlookup –goterms. The output for each gene model was filtered for hits with IPR domains, and from this, we took the length of the longest IPR domain (max IPR) and the total length of all IPR domains (total IPR). InterProScan runs took around 29 h of CPU time.

Metapredict3 [41] was run on the CPU with default options for disorder prediction. Mean disorder was taken for each protein, across individual disorder scores per amino acid.

All other analyses, processing, and visualisations were carried out in Python version 3.11.

Prior to plotting or scoring differences between old and new models, the data from Foldseek was masked based on whether the Foldseek hit had a significant E-value below 0.05. When this threshold was exceeded, the E-value was set to 10 (0 signifies an exact match), and all other Foldseek scores were set to 0. The 0.05 threshold was decided upon by analysing the effect of increasing threshold stringency (Supplementary Fig. S9).

For differences in scores between old and new annotations, where an increase in a score is seen as indicative of an improved gene model, a positive delta (diff) metric will score + 1, and a negative −1. Where a decrease is seen as indicative of an improved gene model, a positive delta (diff) metric will score −1, and a negative +1. The MaxAbs scaling and transformation method from scikit-learn version 1.7.1 [42] was also applied to the differences between old and new models across multiple data sources for analysis and visualisation.

For sums of scores, scores were always summed after applying the MaxAbs transformation. Separate transformations were performed for all 11 variables, AF3 + Foldseek + IPR, AF3 + Foldseek, and AF3 + IPR. The best single variables from each program (*‘AF3 total residue pLDDT over 80 diff.’, ‘Foldseek bitscore diff.’, ‘IPR domain total length diff.’*) were used to calculate sums for AF3 + Foldseek + IPR, AF3 + Foldseek, and AF3 + IPR. Summed scores were then masked with the same win/lose criteria as the previous, where anything > 0 was a win (+1) and <0 was a lose (−1).

## Results

### Overview of manually curated gene structure annotations

Our dataset comprised 444, 317, and 461 gene pairs, respectively, from *Toxoplasma Gondii str. ME49, Fusarium graminearum str. PH-1* and *Aspergillus fumigatus str. Af293*, listed in Supplementary Table S1. We filtered the full gene model sets for pairs with <80% sequence identity to exclude minor changes in coding sequence. Old gene models are a mix of *in silico* and historical manual annotations. New models were exclusively manually curated over a number of months by Böhme.

The manual curation resulted in various kinds of changes to the structural annotation (Supplementary data 1–3). In extreme cases, a gene model was deleted, or was created for a region not previously annotated. In other cases, two gene models were combined or conversely, a single model was split into two distinct coding units. However, in most cases, the change resulted in the inclusion or elimination of one or a small number of exons, a category we call ‘Changed’ - and the focus of this analysis. This category is where we expected a protein structural perspective to be most useful: scores would detect the incompleteness of a structural domain, or the distortion and packing defects resulting from improper incorporation of a translated intron (Fig. 1). Comparing new versus old annotations showed that there was a slight tendency towards longer models post-improvement (Supplementary Fig. S2a), with variable patterns in the numbers of Coding DNA Sequences (CDSs, Supplementary Fig. S2c).

The extent of intrinsic disorder in our three focal species varies considerably, as assessed by Metapredict3 (Supplementary Fig. S2b). For example, the median (of the percentage disorder across each amino acid per protein) across all proteins is ∼20–30% in the new annotations of the two fungi, but much higher at ∼50% in *T. gondii*. Interestingly, the proportion of disordered content is lower after manual curation, especially in *A. fumigatus* (Supplementary Fig. S2b), suggesting that many re-annotations impact disordered regions, potentially challenging our structure-based method.

### Signals from structural prediction

**Figure 2.**
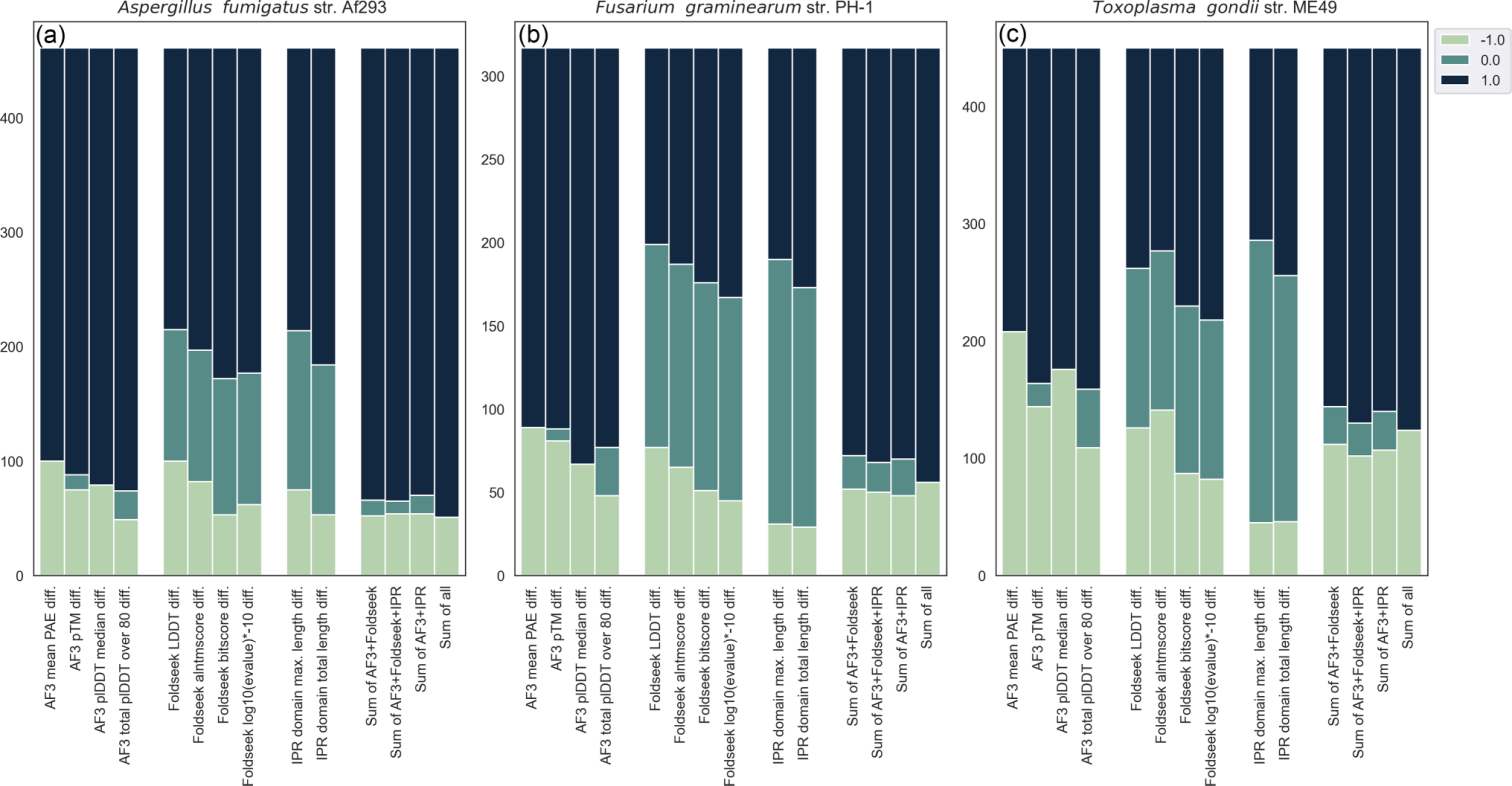
Stacked bars of different values for each score grouped by source (AF3, Foldseek, IPR). Positive changes, i.e. where the score favours the new gene model, are assigned +1, no change is 0, and negative change is −1. The rightmost group of bars in (a), (b), and (c) shows the summed MaxAbs transformed difference scores of combinations of the best AF3 (number of residues with pLDDT > 80), Foldseek (Bitscore), and/or InterProScan (IPR total length) metrics.

**Figure 3.**
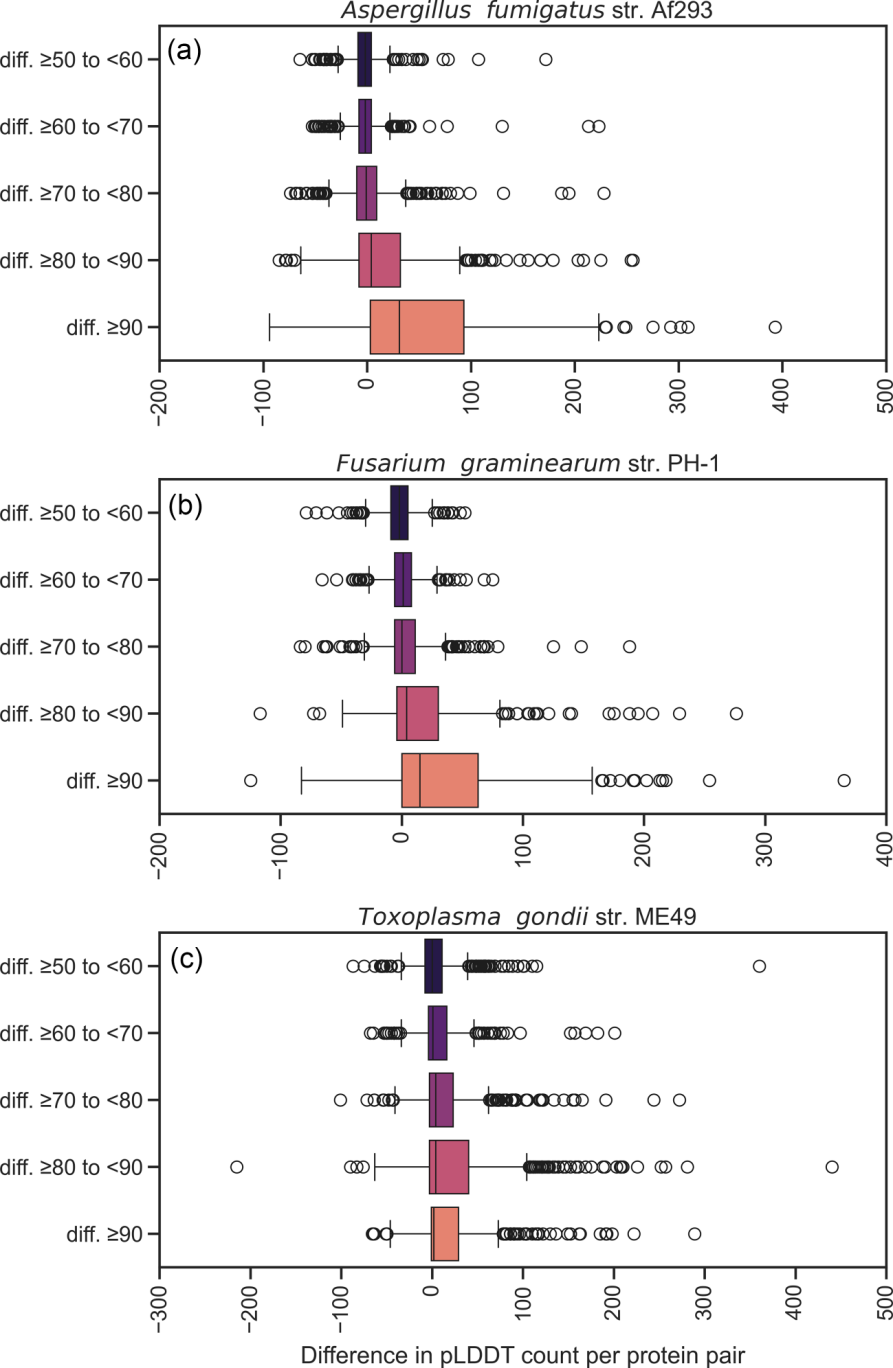
Boxplots of the difference between old and new models in the number of residues that have pLDDT scores within a given range. The box displays the interquartile range (25th percentile, median, 75th percentile), the whiskers represent the 1.5 IQR of the upper and lower quartiles, and beyond that are outliers.

**Figure 4.**
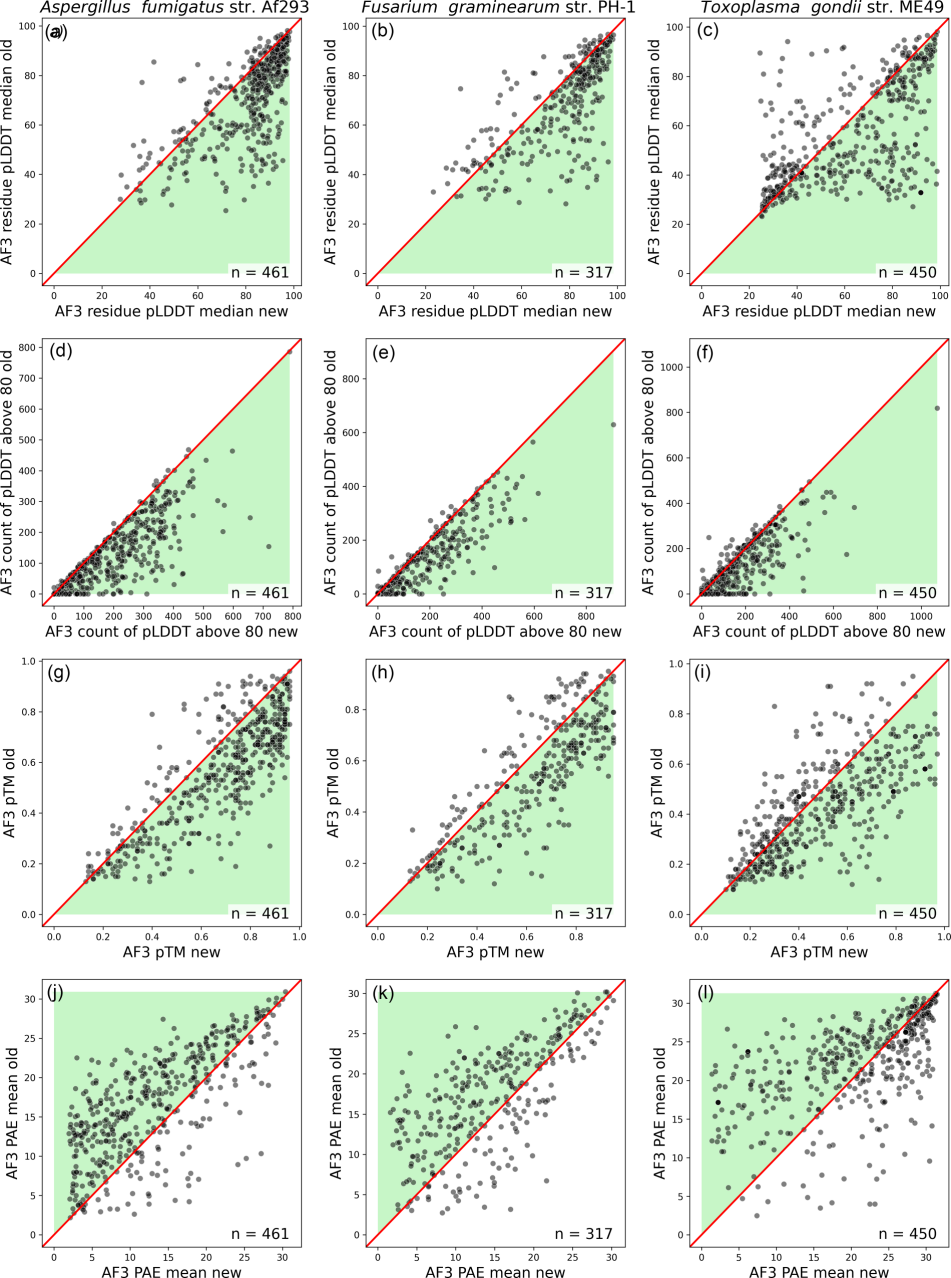
Scatter plots of various AlphaFold 3 scores comparing new models (*x*-axis) to old models (*y*-axis). The shading illustrates the direction of positive change from new to old (e.g. an increase in pTM from old to new is good). The diagonal line represents no change between the old and new models. The number of pairs plotted is displayed in the lower right of each plot.

We explored a variety of scores reflecting structural quality in the AF3 models (see Methods). Median pLDDT, pTM, and mean PAE all preferred the new gene annotations over the old versions (Figs 2–4, Supplementary Fig. S3). Additionally, we found that among the set of AF3-based scores, the number of confidently modelled residues (pLDDT greater than a threshold) provided the clearest signal (Figs 2–4, Supplementary Figs S3 and S4).

In particular, counts of residues that score above 80 pLDDT and above 90 appear to be highly discriminative (Figs 3 and 4, Supplementary Figs S3 and S4). The poor performance of pTM can be explained by its correlation with intrinsic disorder (Supplementary Figs S5 and S6). This aligns with the observation that the pTM of the same structured domain can be depressed by the simple addition of disordered termini [24]. Similarly, the fact that median pLDDT is less discriminatory than the count of high pLDDT residues (Figs 1 and 4) can be explained by the fact that the median will be reduced by the presence of disorder outside of any folded domains, whereas the count will not. We expect structure modelling to offer clear discrimination, principally in the folded structure.

As mentioned, the primary focus here is the gene model pairs exhibiting <80% sequence identity, but analysis of the *A. fumigatus* set of pairs with smaller differences illustrates the persistence of a useful structural signal. Supplementary Fig. S7 illustrates that the count of residues above a pLDDT threshold still typically increases in the structure deriving from the manually curated gene model. Importantly, it also shows that the threshold of 80 is still the best, maximising the number of proteins showing an increase (842) compared to those showing a decrease (244). Supplementary Fig. S8a shows that most of the decreases are small, while other panels of Supplementary Fig. S8 show similar results for other scores.

### Foldseek and InterProScan as supporting tools to AF3

Additionally, useful signal may be gleaned from a model’s similarity to experimentally characterised proteins, independently of raw AF3 scores. We used Foldseek (with the PDB database) and InterProScan to provide several additional scores: LDDT, alntmscore, bitscore, and E-value from Foldseek; IPR domain maximum length and IPR domain total length from InterProScan. It should be noted that the number of IPR domains found does increase from old to new models (Supplementary data 1–3), supporting the idea that there is an overall improvement between datasets.

**Figure 5.**
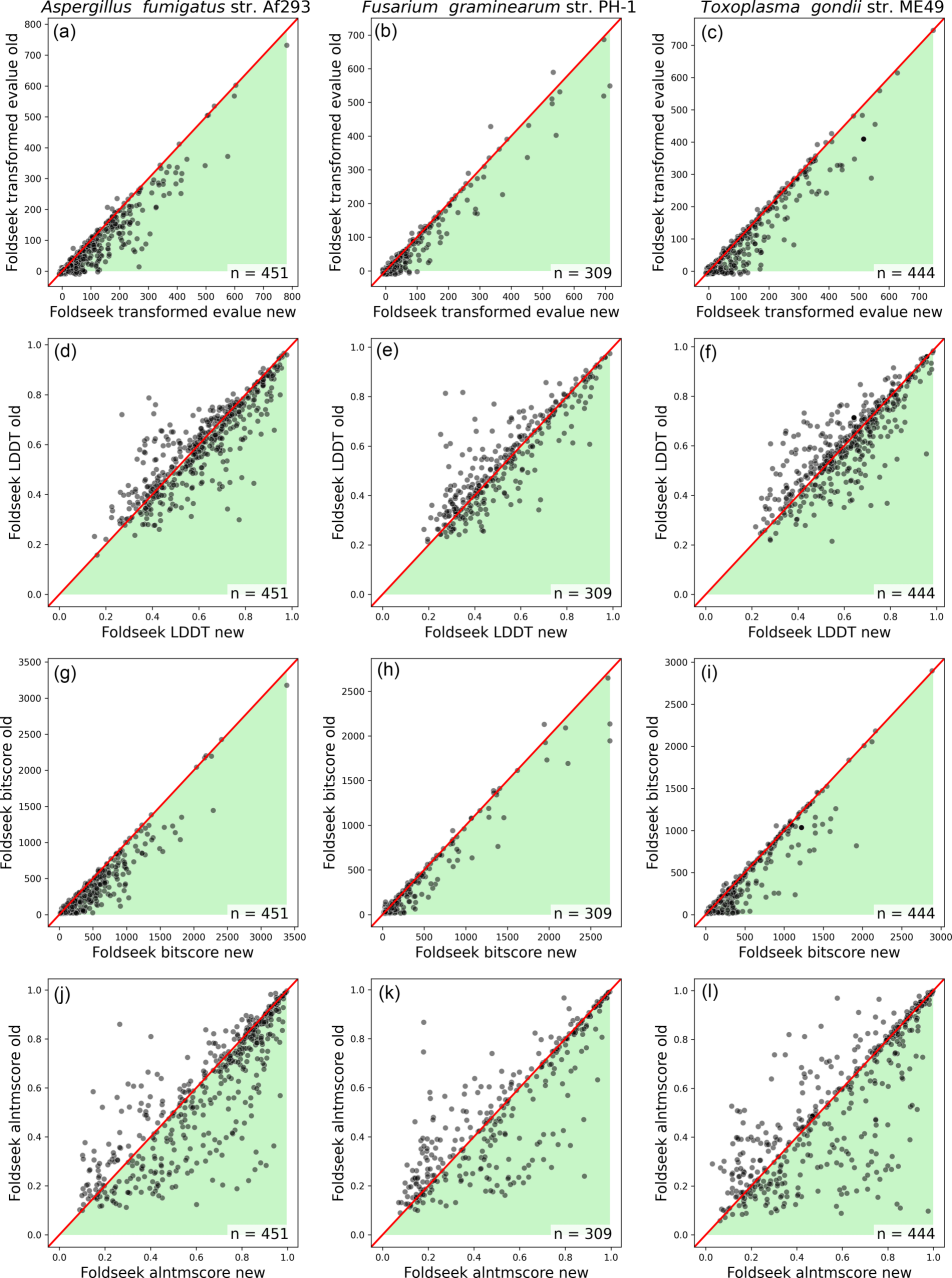
Scatter plots of Foldseek scores comparing new models (*x*-axis) to old models (*y*-axis). The shading illustrates the direction of positive change from new to old. The diagonal line represents no change between the old and new models. The number of pairs plotted is displayed in the lower right of each plot.

**Figure 6.**
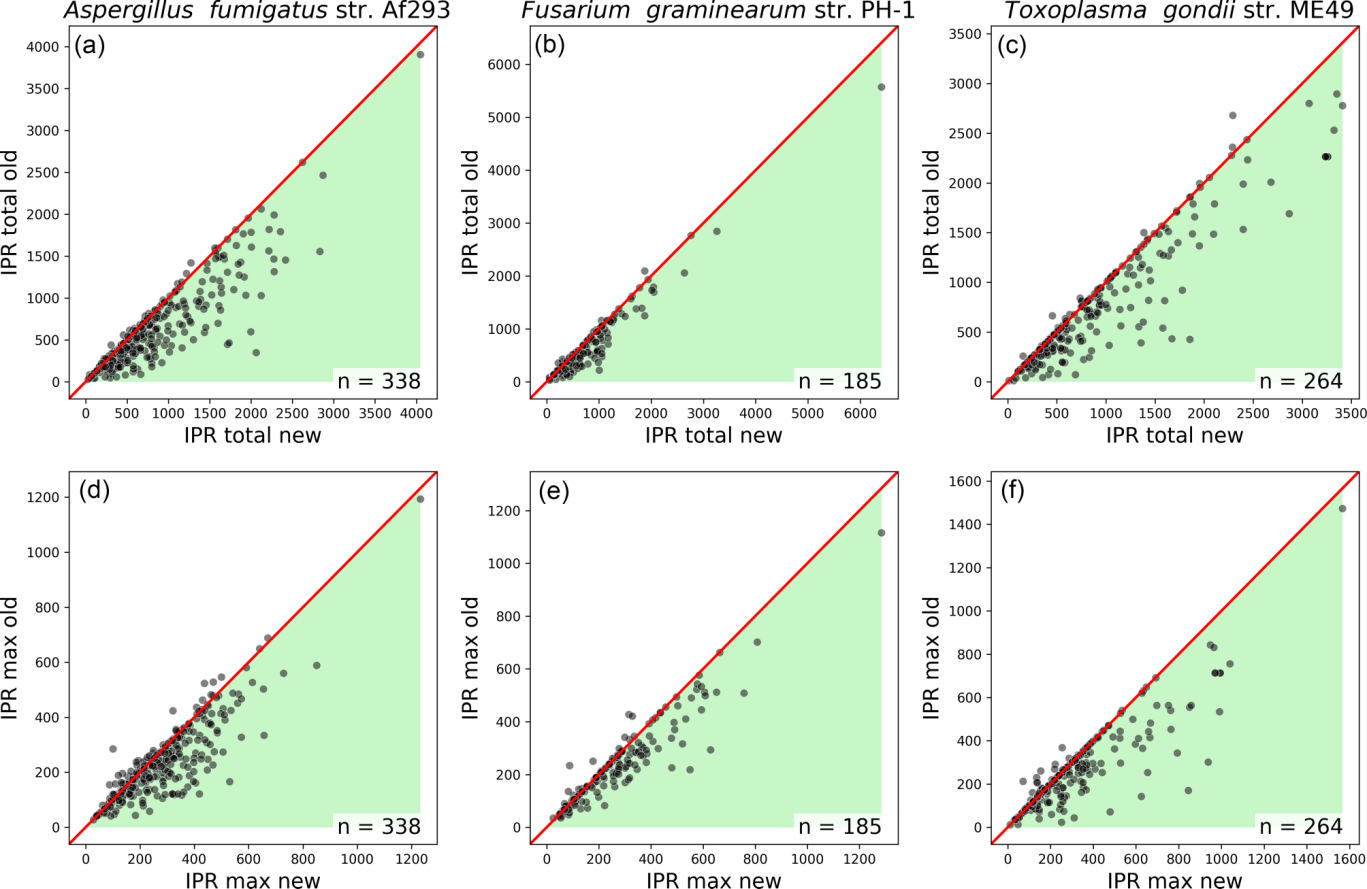
Scatter plots of InterProScan scores comparing new models (*x*-axis) to old models (*y*-axis). The shading illustrates the direction of positive change from new to old (e.g. an increase in IPR; the maximum domain length score from old to new is good). The diagonal line represents no change between the old and new models. The number of pairs plotted is displayed in the lower right of each plot.

Figs 5 and 6 illustrate a general improvement in most scores, though not in Foldseek’s LDDT or, in a consistent fashion across species, in the alntmscore (Supplementary Fig. 9). IPR total domain length is the strongest of these additional signals, but it also provides fewer results per gene pair, with IPR domains missing from a third of all gene models (Figs 5 versus 6). For Foldseek, bitscore appears to be the most reliable signal (Figs 2 and 5), and almost all gene models have a hit. However, many of Foldseek’s top hits are not significant, resulting in a similar distribution of results to InterProScan for our two better annotated species: *A. fumigatus* and *F. graminearum* (Fig. 2a, b, Supplementary data 1–3). Foldseek contributed best to *T. gondii*, which saw larger signals of improvements in its Foldseek scores compared to IPR (Fig. 2c).

### Combining scores

We next wished to assess whether the AF3, Foldseek, and InterProScan scores are redundant, or whether there is a degree of complementarity between them that would encourage integration of all three into future methods. Scatter plots illustrate only modest correlation between the three scores (Supplementary Fig. S10), and heat maps illustrate that, in cases where AF3 fails to differentiate old and new gene models, other scores can provide a signal in favour of the latter (Supplementary Fig. S11).

Taken in combination, we are able to show a general positive trend in the difference in scores between old and new genes (Fig. 2, Supplementary Figs S3 and S12). Fig. 2 shows that combining scores from AF3, Foldseek, and IPR leads to a greater sensitivity to overall model improvement compared to relying on single scores.

We performed ROC analysis (Supplementary Fig. S13) to determine the extent to which scores could differentiate new versus old gene models. The classification was performed by ranking each gene model pair (first or second, for new versus old) based on the sum of maximum absolute scaled values of pLDDT residue count (>80 threshold), FoldSeek bitscore, and InterPro total domain length. Each score pair was then further ranked by the delta in max absolute score between the first and second ranked model, to give the ROC plot and AUC value, counting a true positive if the new model is rank 1, and a false positive if the old model is rank 1.

AUC values were 0.816 (*A. fumigatus*), 0.832 (*F. graminearum*), and 0.816 (*T. gondii*), indicating a strong ability of the scores to classify better from worse gene models, using only three protein-derived scores and without further retraining, for example, using machine learning, to weight particular scores or score combinations more optimally.

### Illustrative cases of success and failure

**Figure 7.**
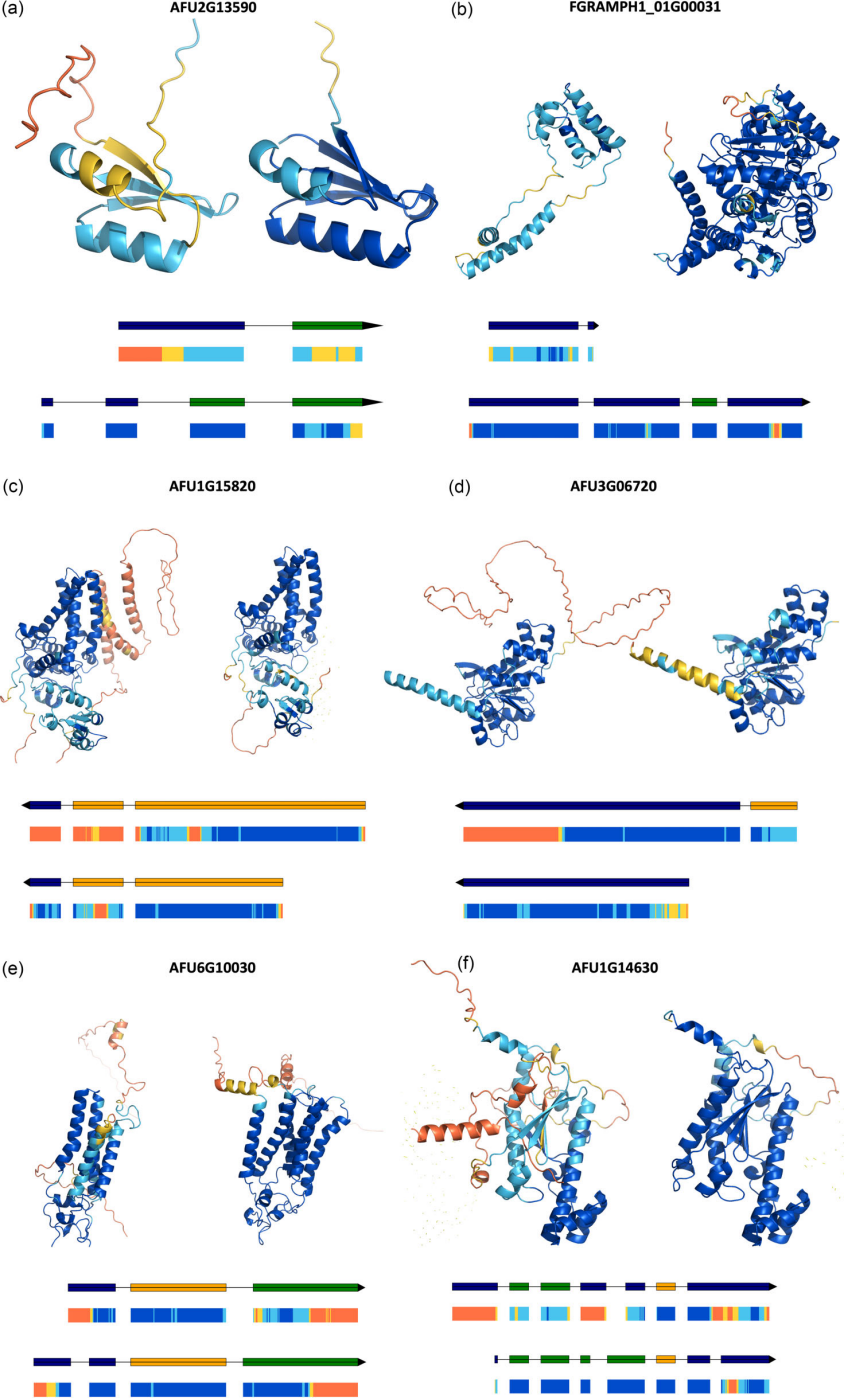
Examples of output protein models for ‘old’ and ‘new’ gene annotations. Each panel shows paired protein models: the ‘old’ model (left) and the corresponding ‘new’ model (right), colored by AlphaFold’s confidence score (pLDDT) from blue (high) to orange (low). Below each pair are schematic representations of the corresponding gene models: the ‘old’ version (top) and the ‘new’ version (bottom). Only coding sequences (CDS) are shown; untranslated regions (UTRs) are omitted for clarity. CDS blocks are coloured according to their reading frame (navy = frame 0, orange = frame 1, green = frame 2), calculated from the GFF phase and lengths of preceding CDS blocks. The black line connects CDS blocks along the genomic locus, with arrowheads indicating the direction of transcription. Differences in the order of CDS colours between the two models indicate potential frameshifts caused by misannotation. A pLDDT ribbon below each schematic shows per-residue confidence along the coding region, using AlphaFold’s standard blue (high confidence) to orange (low confidence) scale. The black line represents the genomic locus, with exon schematics aligned to depict their relative positions. Panels **(a)** and **(b)** illustrate cases where the ‘new’ models demonstrate improved pLDDT, InterProScan, and Foldseek scores. Panels (**c–f**) show a minority of cases where one or more scores decrease. Cases are discussed in the main text.

The bare numbers in Figs 2 –6 indicate the value of the scores introduced here, but we additionally illustrate typical successes in Fig. 7a and b. In these examples, the ‘old’ sequences were derived from misannotated genes containing errors such as missing exons, frameshifts, or incorrect start sites. The corrected annotations in the ‘new’ models yield accurate protein sequences, restoring structural integrity and completing domain matches.

Interestingly, the example in Fig. 7a includes a structural element that is in common between the old and new models, but which corresponds to different sequences in the two cases. Structural alignment of the two models reveals that they are sequence-identical only from residue 20 of the new model, corresponding to residue 31 of the old model. The distinct sequence prior to this region—resulting from the original model being incorrectly predicted as a two-exon gene, as opposed to the new model, where it is predicted as a four-exon gene—folds to include a superimposable β-strand. The explanation seems to be that AF3, recognising the expected fold from the rest of the sequence, understands that a β-strand should be present and forms it from the most suitable available sequence. However, not being the correct sequence, the strand is recognised as of low quality and receives low pLDDT scores. Notably, the neighbouring helix in the structure, which *is* composed of the same sequence, is also low-confidence in the old model since the β-strand mentioned above fails to provide a favourable environment for it. Thus, this example illustrates a magnifying effect by which the number of high-confidence residues in the model of an incorrect gene model can be reduced both in the primary region - a translated intron, a frame-shifted translation - but also in neighbouring parts of the structure, enhancing the signal that favours the correct sequence.

In general, the AF3, Foldseek, and IPR scores correlate, albeit weakly, with *r*^2^ values between 0.23 and 0.40 (Supplementary Fig. 10), but Fig. 7 also illustrates cases of discordance between the scores and what can be learned from them. In Fig. 7c, the number of high-confidence residues slightly decreases (from 299 to 288), but the core structure remains accurate, with higher Foldseek and InterProScan scores reflecting better correspondence to known folds and domains, possibly due to more canonical sequence coverage. In Figure 7d, all three metrics decrease slightly (high-confidence residues drop from 229 to 200; Foldseek from 136 to 134; InterProScan from 817 to 762). The sequence difference between old and new annotations is simply an erroneous extra stretch of 106 residues at the N-terminus of the former. Here, the unexpected lower confidence for the helix that starts the correct protein sequence may result from differences, induced by the flanking sequence, in the Multiple Sequence Alignment (MSA) generated for the two cases: it is known that MSA depth is reflected in pLDDT values output by AlphaFold [43]. The lower confidence in the C-terminus may also reflect MSA differences or could simply derive from the natural variability sometimes present between models. Importantly, Fig. 1 illustrates how pLDDT values do not respond to MSA depth alone: sequentially identical neighbouring residues of the introduced loop drop in confidence (cyan left, blue right) despite the MSAs generated during modelling of each having similar depth (Supplementary Fig. 1).

In Fig. 7e, both high-confidence residue count and InterProScan score increase, while Foldseek decreases slightly (from 41 down to 34). The proteins matched by FoldSeek are similar, so it appears that the missing helix in the old structure leads to a distortion away from the consensus helical packing of the family. Finally, in Fig. 7f, the number of high-confidence residues and the Foldseek score both increase, while the InterProScan score decreases slightly (1173 down to 1113). Despite being manually curated and biologically complete, the ‘new’ sequence may show slightly shifted alignments or small insertions/deletions (e.g. loops or linkers) relative to the statistical domain models used by InterProScan. These subtle differences can lower sequence-based alignment scores even when the overall structure is more accurate.

One important conclusion from examination of these cases is that discordant scores typically change in the wrong direction by only a small amount, suggesting that consideration of the magnitude of the change in a score, not just its direction, should be given more attention in the future. This reflects the fact that all the scores here will be susceptible to some noise.

### Protenix-Mini scores are as useful as those of AF3

**Figure 8.**
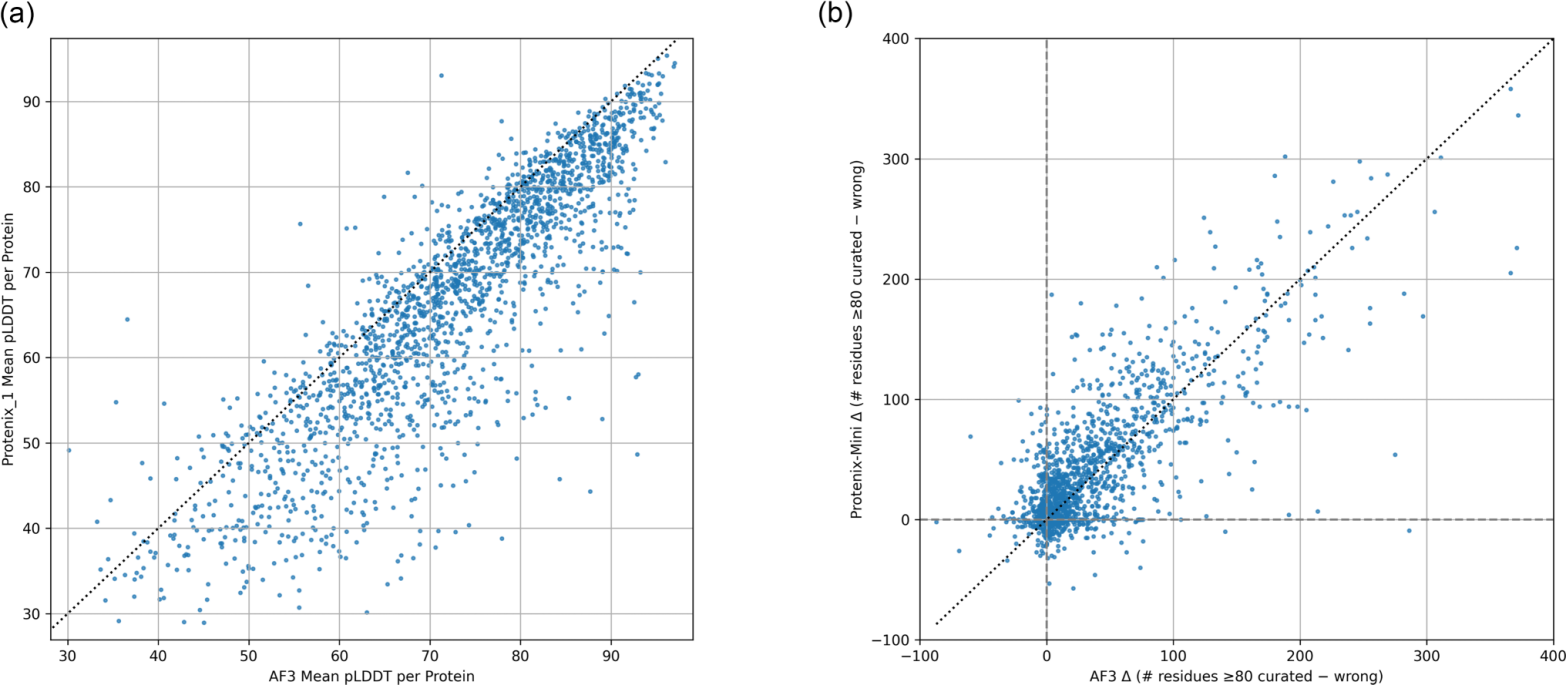
Scatter plots for comparison of AF3 and Protenix-Mini scores across all three species (**a**) Mean pLDDTs species and (**b**) Differences in numbers of residues with pLDDT > 80 (positive meaning higher in the curated version). Points above the diagonal are those where the delta pLDDT is higher for Protenix-Mini.

Although highly efficient compared to previous generations of software, AF3 is still quite computationally demanding, and the environmental implications of proteome-wide structure prediction should not be ignored. Accordingly, we have been exploring Protenix-Mini, which simplifies various elements of the calculation and, in particular, substitutes the use of a language model for the compute-intensive MSA generation, thereby dramatically reducing compute requirements for very modest drops in prediction accuracy [39]. Mean Protenix-Mini pLDDT values show a similar distribution to those of AF3 (Fig. 8a) and, crucially, the differences in scores calculated from Protenix-Mini outputs are just as diagnostic of gene model accuracy as those derived from AF3 predictions (Fig. 8b). Recent efforts have been directed towards increasing the efficiency of the AF3 MSA generation step, e.g. [44] yet Proteinx-Mini is much faster than AF3, even when the latter is supplied with precomputed MSAs. On the set of 461 *A. fumigatus* proteins, the timings are 1 h for Protenix-Mini versus 43 h for AF3 on the same machine. With GPU requirements as low as one second for proteins shorter than 200 residues, the use of Protenix-Mini should enable the greener and more efficient rollout of our newly developed scores.

## Discussion

Manual curation by experts actively improves gene structure models, but it is well known to be a time-consuming and expensive task [3, 45]. Building on previous work confined to human isoforms [19], here we explore the idea that scores from 3D structural prediction software could be a useful tool in improving automated gene model quality scoring. These scores could ultimately be used in improving gene annotation quality for many new genomes that are sequenced each year, which will likely never see manual curation (https://www.ncbi.nlm.nih.gov/genbank/statistics/).

Overall, while protein structure-based scores are clearly powerful, our results support the established wisdom that there is no single method of determining the quality of all gene or protein models [46]. The magnitude of the scores is also clearly important since we show that small changes in the wrong direction can arise by chance. We chose to focus on AlphaFold 3 and combine its scores with Foldseek (versus the PDB) and InterProScan results to complement pure model-based scores with those derived from a comparison to known protein structural domain data. AlphaFold 3 scores largely support the manual changes made by our curator, and these are further enhanced when combined with other structural information from both Foldseek and InterProScan. InterProScan is powerful on its own, but is limited in scope by the fact that only two-thirds of the gene models have a significant hit with an InterPro domain. Similarly, Foldseek was only informative for folds currently observed in the PDB. Future work could explore extending the search to TED (The Encyclopedia of Domains; [47]), which captures and classifies recurring domains in the AlphaFold Protein Structure Database [48]. The risk of circular logic, by which a protein could be matched to a model of a protein derived from an incorrect gene model, could be minimised by using the newly developed q-score, which favours domains that are more ordered and which are seen repeatedly across the database.

One confounding issue identified early on was the impact of intrinsic disorder. Scores like pTM correlate with disorder (Supplementary Fig. 6), rendering our analyses less likely to define a preferred gene model where differences between old and new gene models fall in disordered regions (although interaction motifs can manifest as islands of higher pLDDT in disordered regions [49]). Among future avenues to explore for disordered regions are the use of a protein language model, as employed by the NetStart 2.0 package for translation start prediction [50]. Other approaches could exploit the observation that disordered regions can be annotated with different ‘flavours’, each with distinct functional correlations [51]. The simple observation that a protein region has a strong ‘flavour’ may well be a significant sign that it is genuinely translated sequence, but it may also be possible to assess the congruence of the functions of the disorder flavour and the structural domains found in the same protein.

As most gene finders produce multiple plausible gene models per locus (based on RNASeq read alignment or *ab initio* signals), we envisage that a classification and ranking tool can be developed incorporating signals from structure prediction and structure searching, complementing domain prediction (e.g. InterProScan) and other protein-based analyses (e.g. PSAURON). Gold-standard sets of curated gene models, such as those described here, can be used for training a machine-learning-based classifier to assign optimal weights to different scores: as we have demonstrated with ROC analysis, even without training, there is a strong signal for differentiating improved models. The much quicker, yet equally useful modelling enabled by Protenix-Mini should also encourage the incorporation of scores derived from structure predictions.

Overall, despite challenges presented for some particular proteins (such as intrinsically disordered), we have demonstrated strong signals and complementary signals from protein structure tools, which can be adopted to aid future software development. The attraction of tools that work directly from a protein fasta file is that these can also help with gene model scoring, in the absence of deep experimental data (e.g. transcriptomics). We envisage that protein structure-derived metrics can be added to improve gene finding pipelines or post-processing tools that select the best gene model from a panel of alternatives.

## Conclusions

In conclusion, scores derived from the latest structural prediction tools can be highly effective for the selection of gene models, but have room for improvement. Although further work exploring other species is required, combining the outputs of structural prediction tools and gene accuracy measures may be useful in producing quality scores for gene models in general. It will require concerted effort to curate gene model databases for deep learning models that capture the width and breadth of variation in nature, and databases like VEuPathDB that integrate multiomics data are well placed to support this work.

Code for reproducing the analyses here is in GitHub: https://doi.org/10.5281/zenodo.17379945.

## Supplementary Material

gkag369_Supplemental_Files

## Data Availability

Data used in this publication can be found at FungiDB. The datasets generated or analysed in this study, along with the code used, can be found on Zenodo with the following DOIs: 10.5281/zenodo.17287464, 10.5281/zenodo.17290574, 10.5281/zenodo.17290584
