# Supplementary material for: The promise of AlphaFold for gene structure annotation
Source: Nucleic Acids Res. 2026 Apr 24;54(8):gkag369. doi: 10.1093/nar/gkag369 (PMC13107130; doi:10.1093/nar/gkag369)
Supplement: gkag369_Supplemental_Files [file gkag369_supplemental_files.zip › Davison et al Supplementary.docx]

Supplementary information for “The promise of AlphaFold for gene structure annotation”

Helen Rebecca Davison^1^, Ulrike Böhme^1^, Shahram Mesdaghi^1,2^, Paul A. Wilkinson^1^, David S. Roos^3^, Andrew R. Jones^1^, Daniel J. Rigden^1^

#

# Institution addresses

^1^ Department of Biochemistry, Cell and Systems Biology, Institute of Systems, Molecular and Integrative Biology, University of Liverpool, United Kingdom

^2^ Computational Biology Facility, University of Liverpool, United Kingdom

^3^ Department of Biology, University of Pennsylvania, Philadelphia, PA 19104, USA

# Supplementary data

**Supplementary data 1**, *Aspergillus fumigatus* data files (AlphaFold 3, Metapredict3, InterProScan, Foldseek, fasta, gff, and summary of structural changes) - 10.5281/zenodo.17287464

**Supplementary data 2**, *Fusarium graminearum* data files (AlphaFold 3, Metapredict3, InterProScan, Foldseek, fasta, gff, and summary of structural changes) - 10.5281/zenodo.17290574

**Supplementary data 3**, *Toxoplasma gondii* data files data files (AlphaFold 3, Metapredict3, InterProScan, Foldseek, fasta, gff, and summary of structural changes) - 10.5281/zenodo.17290584

# Supplementary Methods

### Structural Annotation of *Toxoplasma gondii* ME49, *Aspergillus fumigatus* Af293 and *Fusarium graminearum* PH-1.

Manual structural curation of gene models was performed using the Apollo graphical browser-based curation tool (version 2.6.7) [(1)](https://paperpile.com/c/Ef4hxz/kPoUp) integrated within VEuPathDB. Gene models were examined using RNA-Seq evidence tracks available in VEuPathDB to refine structural features, including exon-intron boundaries, start codon positions, and isoform annotations. Additional edits involved merging or splitting gene models and adding new gene models. BLAST [(2)](https://paperpile.com/c/Ef4hxz/Giz28) and InterPro [(3)](https://paperpile.com/c/Ef4hxz/HlzaW) results were not used at this initial curation stage. Furthermore, at no point did the curator make reference to any protein structural information, experimental or predicted.

Once structural annotation in Apollo was finalised, a patch build process was initiated. The first process that happens is the gene model diff script is initially run (<https://github.com/VEuPathDB/gene_model_diff>) on both, the core database of the original gene set and the dumped apollo annotation file. Only models that are tagged with a finished status are parsed through. The gene model diff provides an initial quality control mechanism that flags problematic annotations (models that are devoid of start and stop codons against the logic of a gene model) and summarises the changes whether models are new, splits, merges, modified or the same. During the patch build process split, merged and new genes will get a new gene/transcript ID.

[1. Lee,E., Helt,G.A., Reese,J.T., Munoz-Torres,M.C., Childers,C.P., Buels,R.M., Stein,L., Holmes,I.H., Elsik,C.G. and Lewis,S.E. (2013) Web Apollo: a web-based genomic annotation editing platform. *Genome Biol.*, **14**, R93.](http://paperpile.com/b/Ef4hxz/kPoUp)

[2. Altschul,S.F., Madden,T.L., Schäffer,A.A., Zhang,J., Zhang,Z., Miller,W. and Lipman,D.J. (1997) Gapped BLAST and PSI-BLAST: a new generation of protein database search programs. *Nucleic Acids Res.*, **25**, 3389–3402.](http://paperpile.com/b/Ef4hxz/Giz28)

[3. Blum,M., Andreeva,A., Florentino,L.C., Chuguransky,S.R., Grego,T., Hobbs,E., Pinto,B.L., Orr,A., Paysan-Lafosse,T., Ponamareva,I., *et al.* (2025) InterPro: the protein sequence classification resource in 2025. *Nucleic Acids Res.*, **53**, D444–D456.](http://paperpile.com/b/Ef4hxz/HlzaW)

# Supplementary tables

Supplementary table 1. A summary of changes in genes between versions.

Some genes may have multiple changes (e.g. has been changed and merged). Splits: one gene that has now been split into two genes. Merges: two, three or four genes now merged into one, due to previously unrecognised introns. changed: adding/deleting exons, changing exon/intron boundary, translational start shifted downstream, translational start shifted upstream, or corrected from an incorrect frame. Isoforms: an additional transcript has been added.

|  | ***Toxoplasma gondii*** **str. ME49** | ***Fusarium graminearum*** **str. PH-1** | ***Aspergillus fumigatus*** **str. Af293** |
| --- | --- | --- | --- |
| Total genes from the new annotation | 1321 | 2564 | 2430 |
| Total genes from the old annotation | 1461 | 2260 | 2140 |
| **Modification type incidence** |  | | |
| merge | 141 | 43 | 48 |
| Isoform gain | 30 | 16 | 4 |
| changed | 1151 | 2047 | 1969 |
| split | 18 | 113 | 36 |
| deletion | 269 | 39 | 86 |
| new | 262 | 343 | 376 |

# Supplementary figures

1.
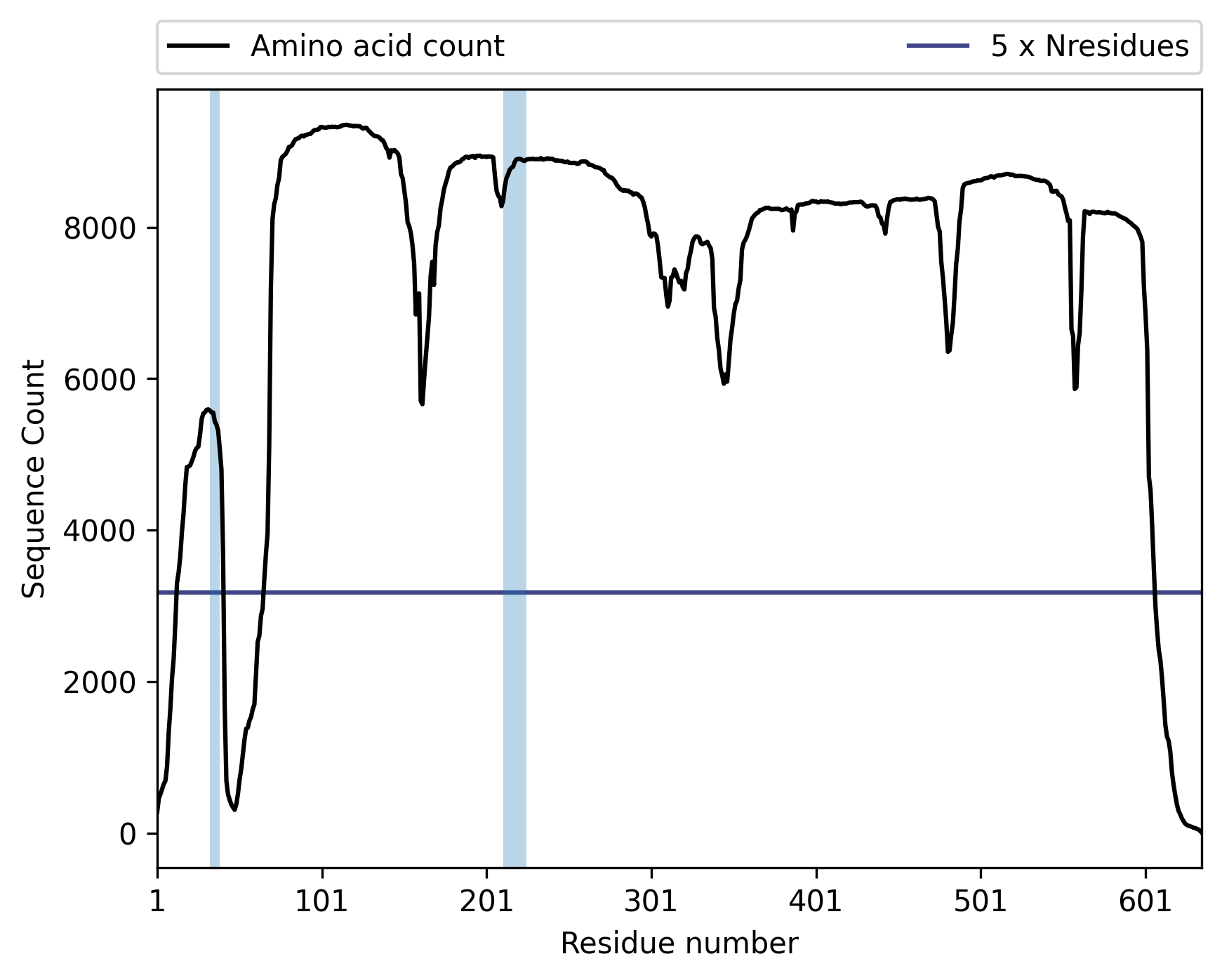

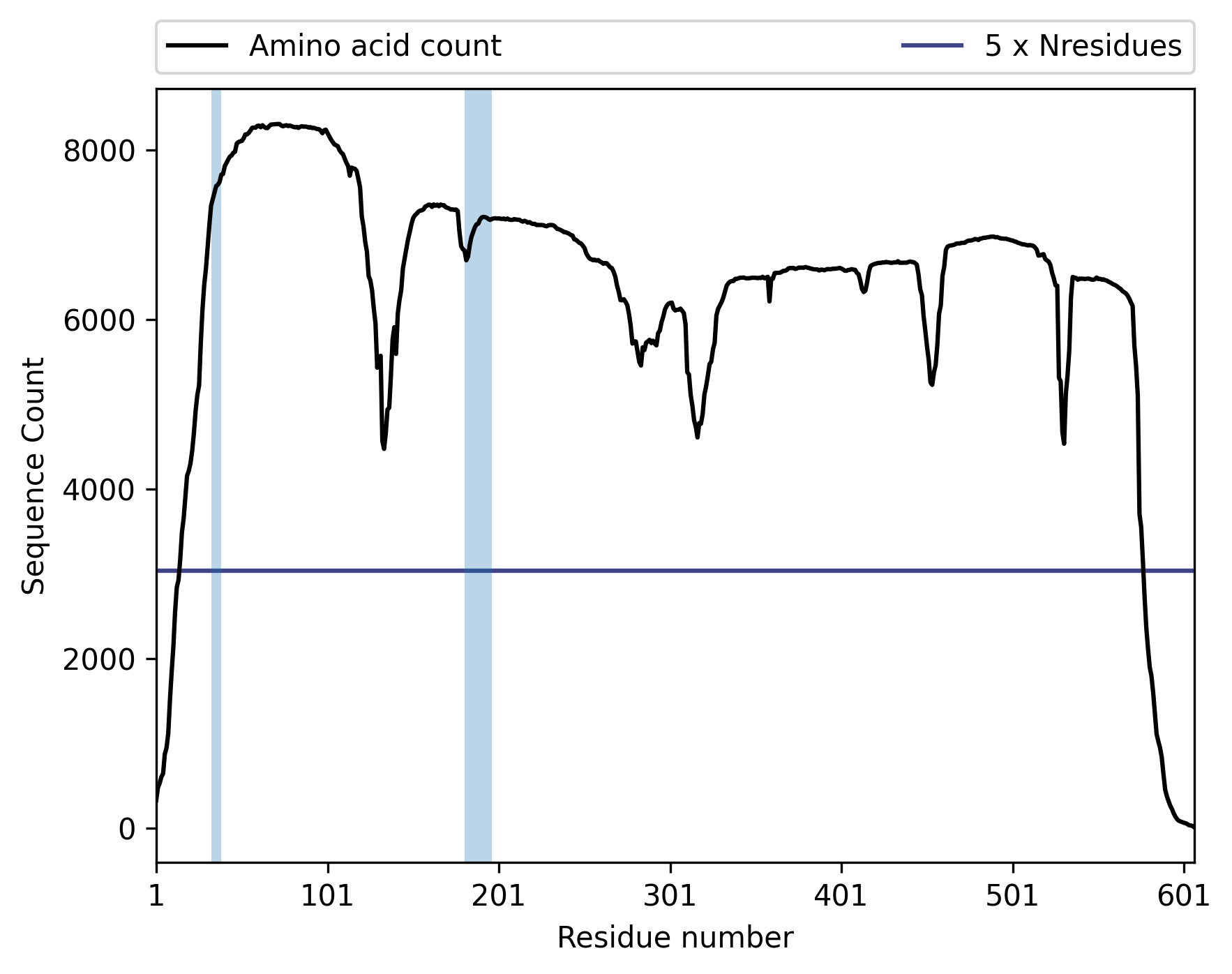

   1. OsNip_10G014190_01 Os10t0469900-01
2. ***Supplementary figure 1. MSA depth for the structures corresponding to gene models shown in Fig 1.*** *The blue shaded regions (33-39, 209-225 in OsNip_10G014190, 33-39, 181-197 in Os10t0469900) are identical sequences in the two models with very similar MSA depths (actually greater in the wrong gene model) but which have lower confidence (cyan) in the incorrect gene model (left in Fig 1) but higher confidence (blue) in the correct gene model (right)*
3.
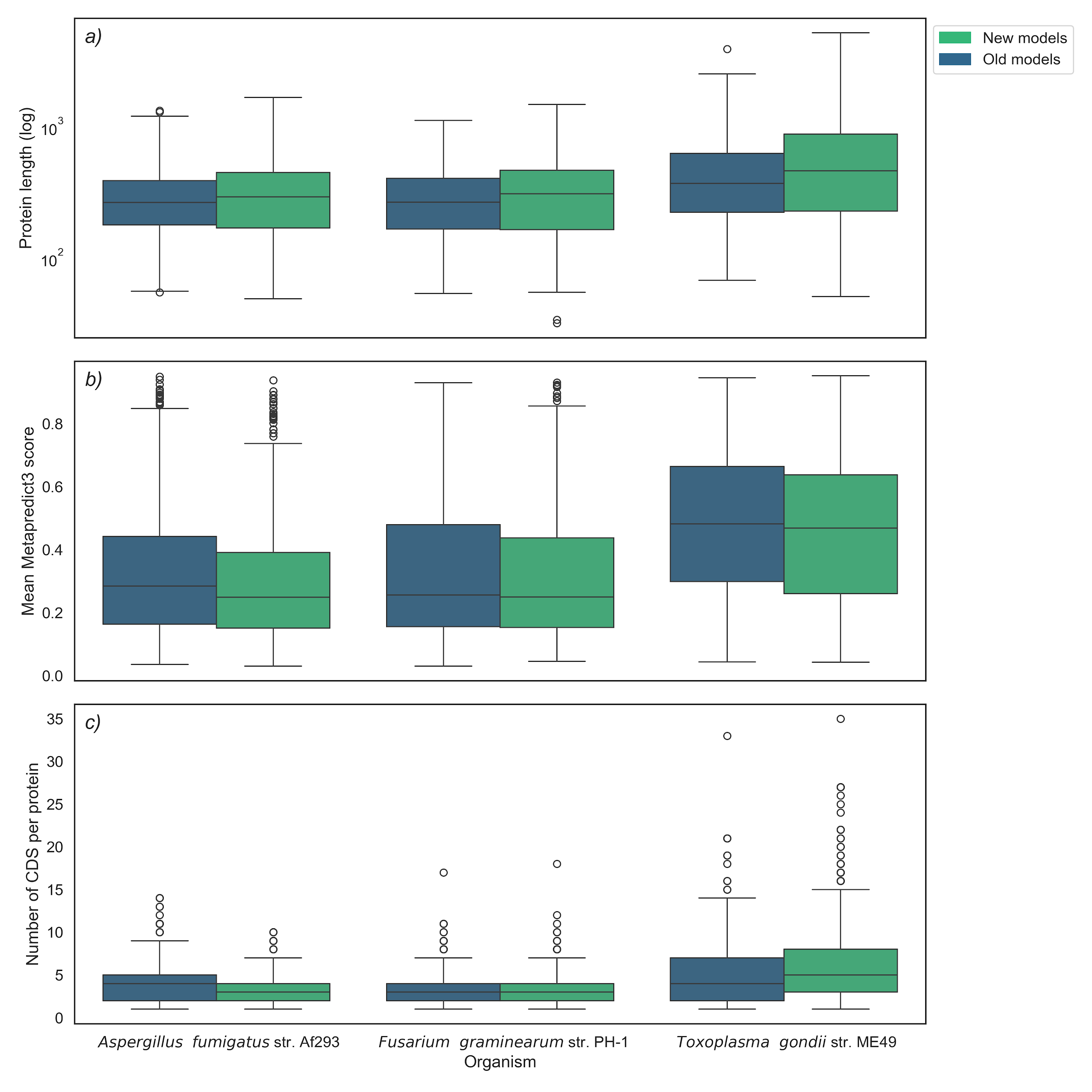

4. ***Supplementary figure 2. Descriptive summary comparison between old and new annotations for all three species,*** *illustrating the change in a) protein length, b) mean Metapredict3 score, and c) the change in the number of coding DNA sequences (CDS) per protein.*
5. *
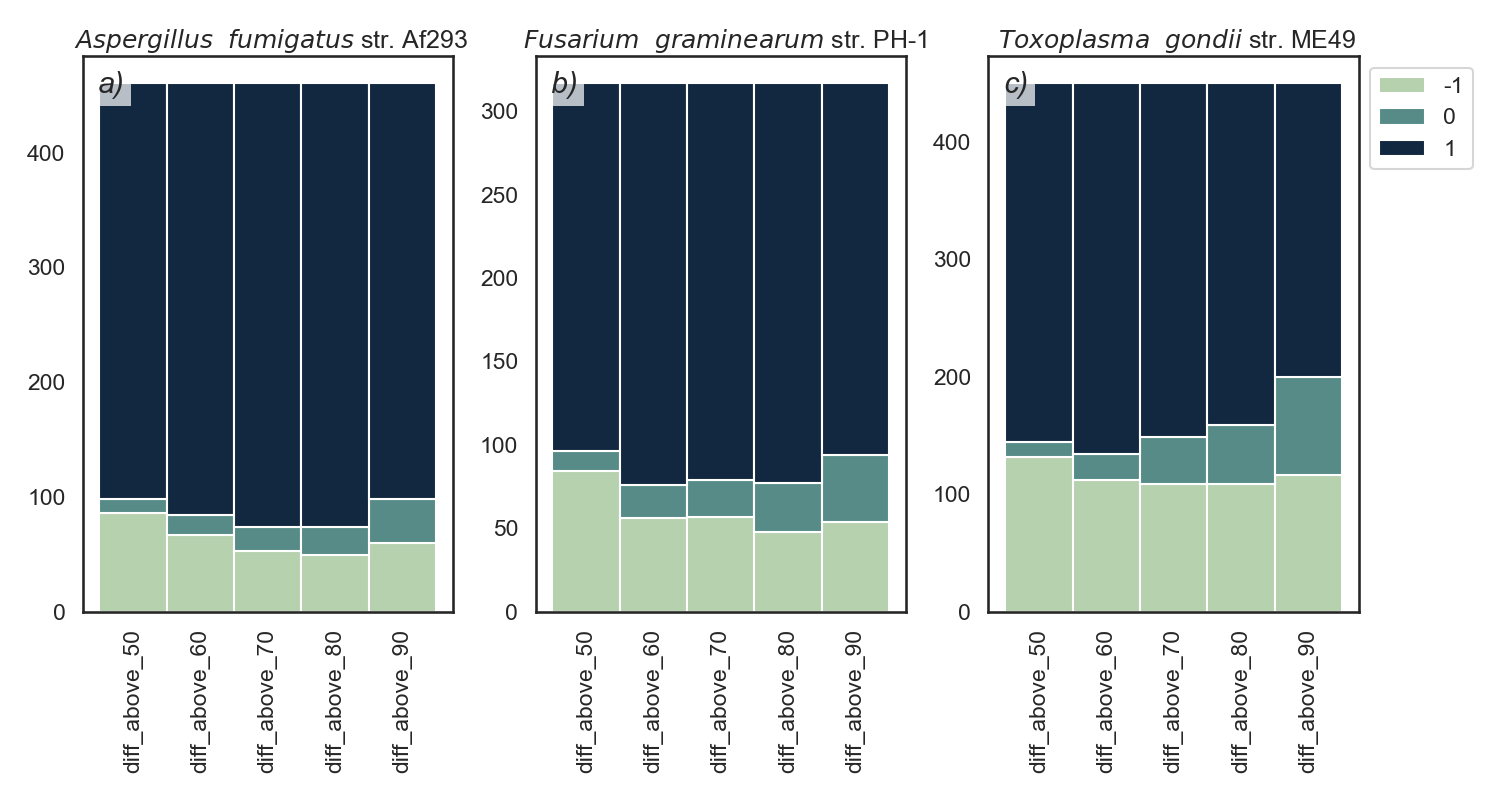
*
6. ***Supplementary figure 3. Win/loss stacked bars for pLDDT counts above a threshold.*** *A win (+1) shows where a new model has more pLDDT residues scoring above the threshold listed on the x axis. A draw (0) is where the number of pLDDT residues scoring above the threshold are the same. A loss (-1) is where the new model has fewer pLDDT residues scoring above the threshold.*
7.
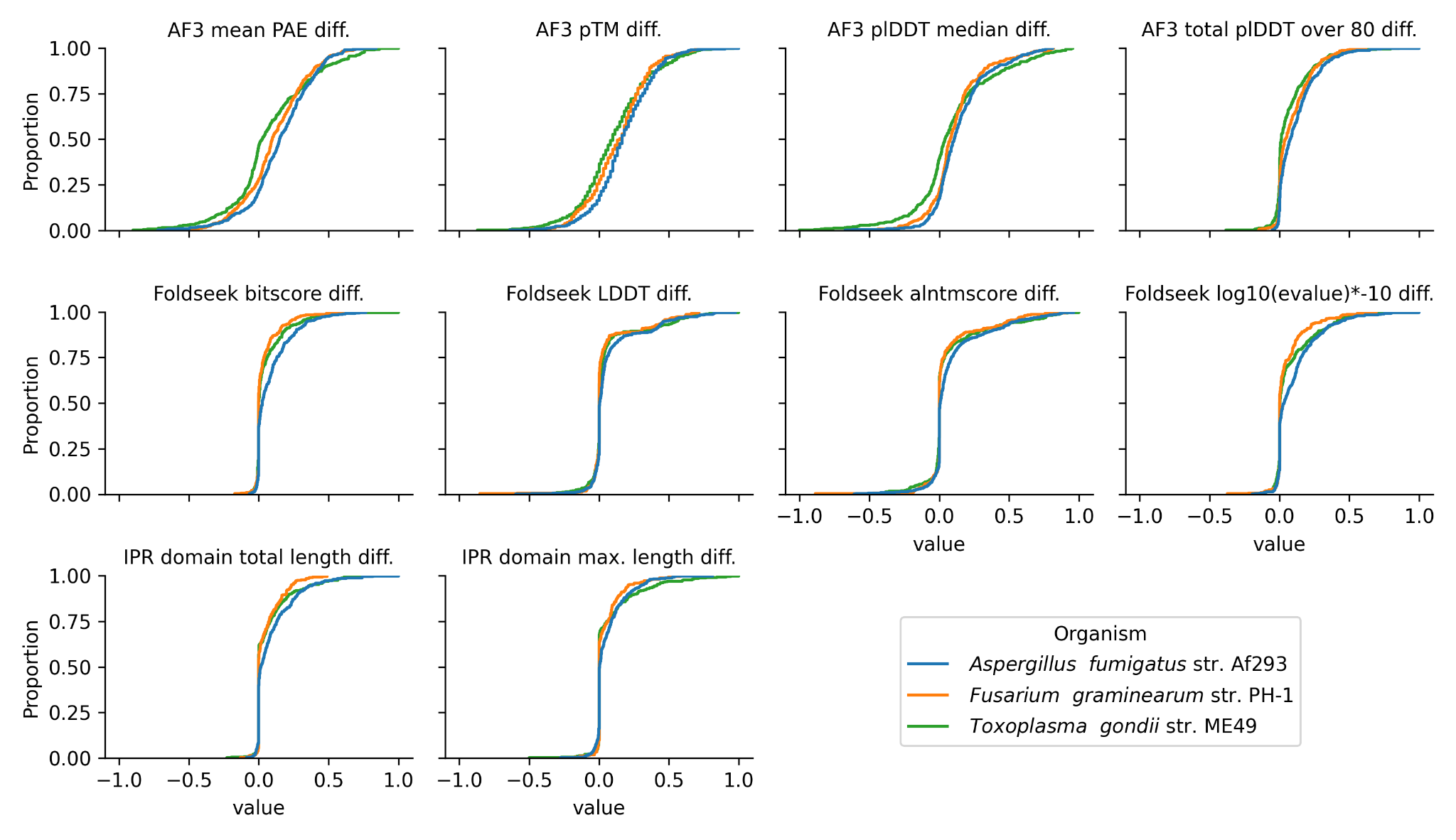

8. ***Supplementary figure 4. Empirical Cumulative Distribution Function (ECDF) plots for the various scores explored here***

***
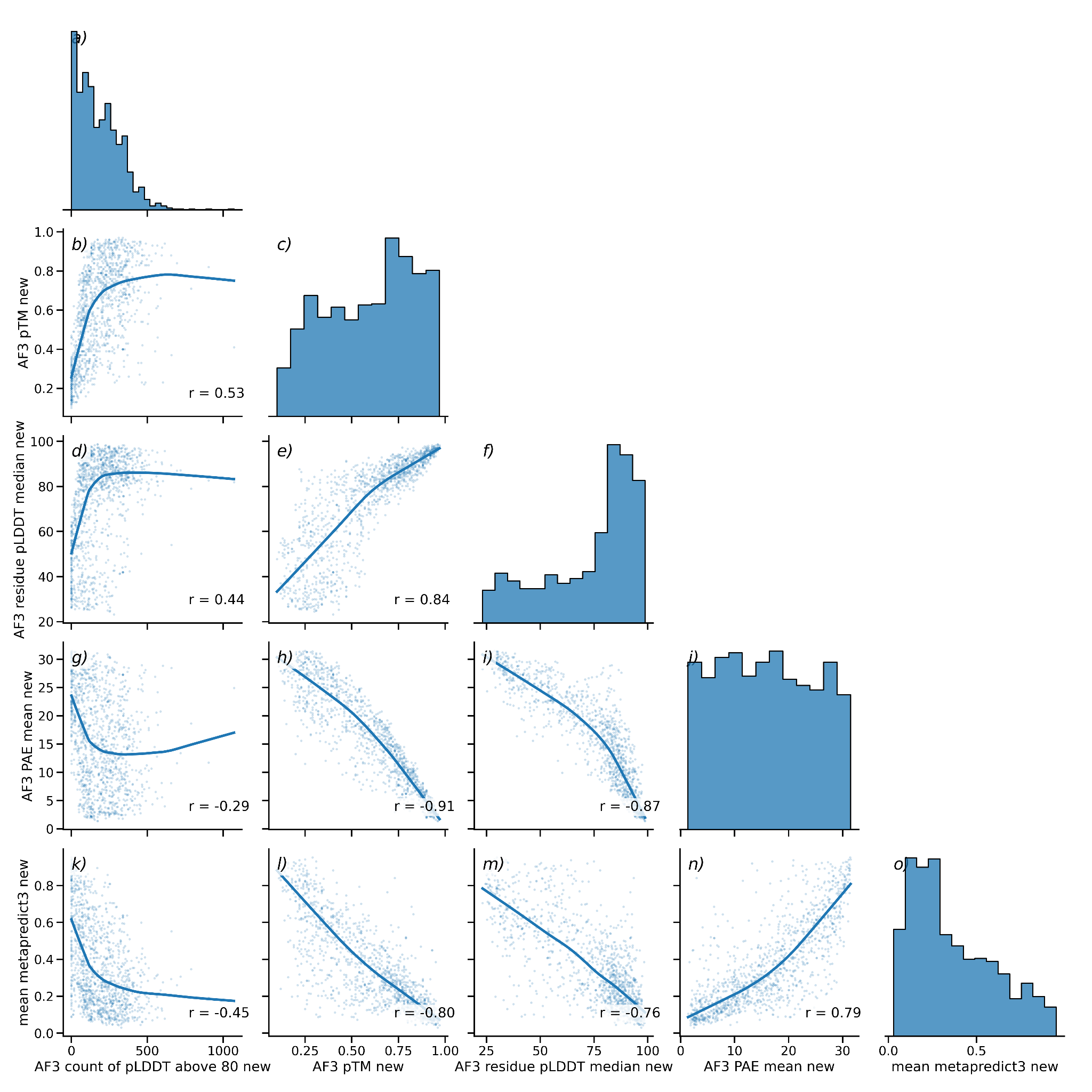
***

***Supplementary figure 5. Paired scatter plots of mean Metapredict3 disorder scores, AF3, PAE, pTM, pLDDT median, and pLDDT > 80.*** *Scores across all three species for new models are shown. A LOWESS regression line is shown in solid blue, and a Pearson's r score is displayed for each correlation. A histogram for each of the five scores is shown on the diagonal.*


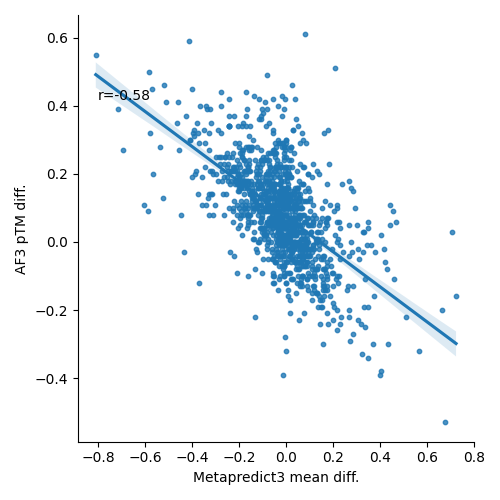


***Supplementary figure 6. Correlation of differences in change of disorder prediction and changes in AF3 pTM.*** *Where the pTM difference is positive, the structure prediction for the curated gene model has a higher pTM than that of the original model. Such cases tend to have a very negative difference in Metapredict3 predicted intrinsic disorder i.e. the translation of curated gene model is predicted to be much less disordered than that of the original.*


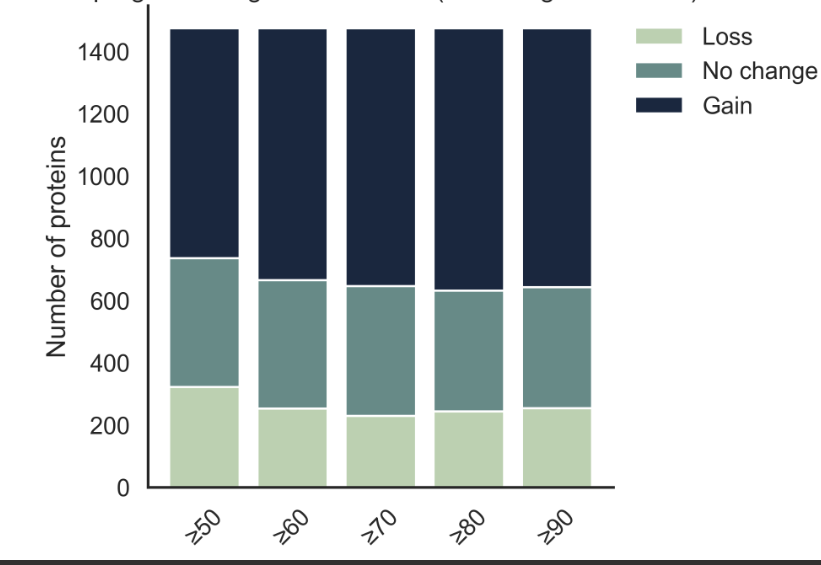


***Supplementary figure 7 Analysis of more similar* A fumigatus *protein pairs exhibiting >80% sequence identity: stacked bars of difference values for counts of residues above the given pLDDT thresholds.*** *Positive changes i.e. where the score favours the new gene model, are assigned +1, no change is 0, and negative change is -1.*

*
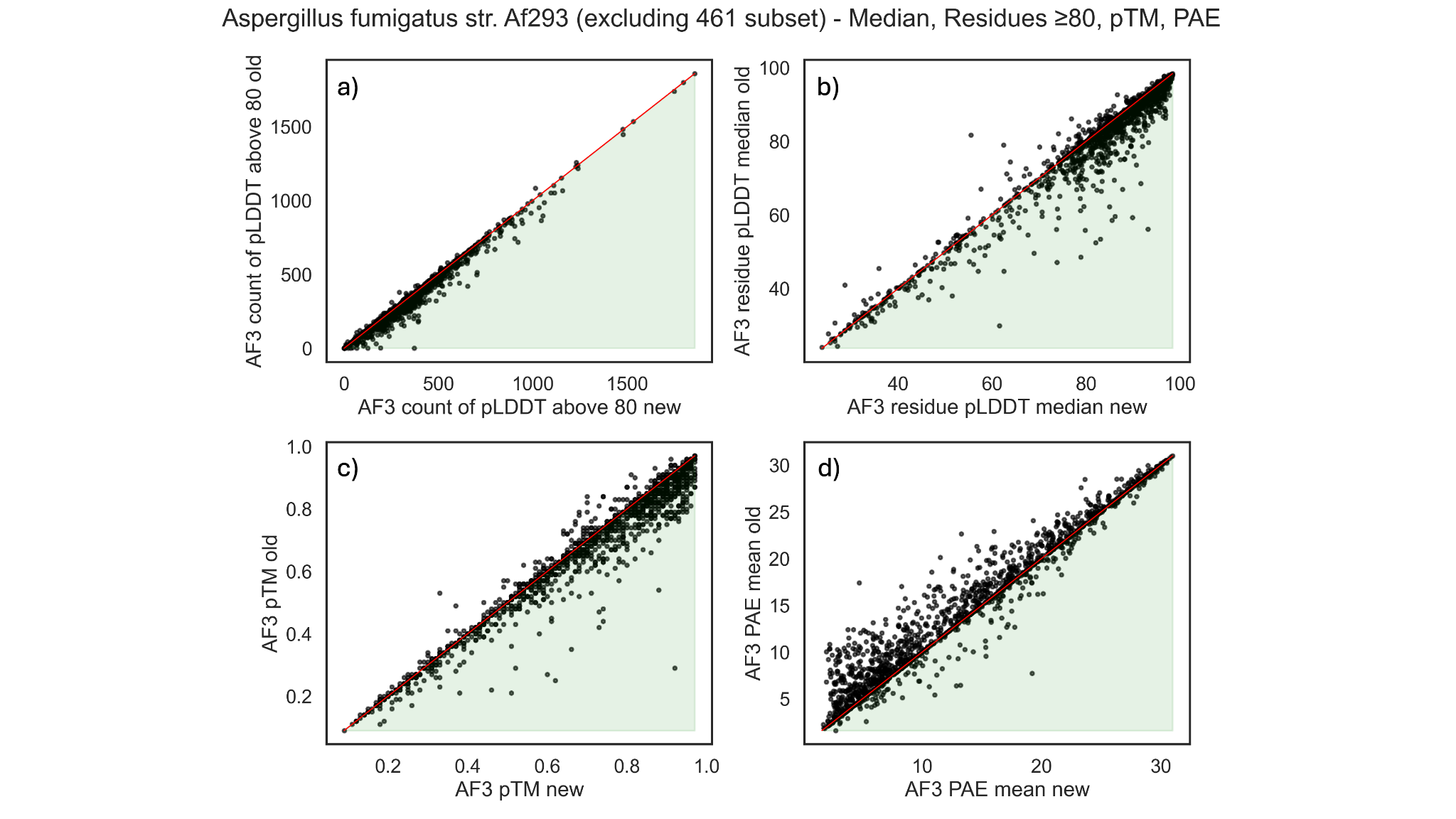
****Supplementary figure 8. Analysis of more similar* A. fumigatus *protein pairs exhibiting >80% sequence identity: Scatter plots of various AlphaFold 3 scores comparing new models (x-axis) to old models (y-axis).*** *The green shading illustrates the direction of positive change from new to old. The diagonal red line represents no change between old and new models.*

*
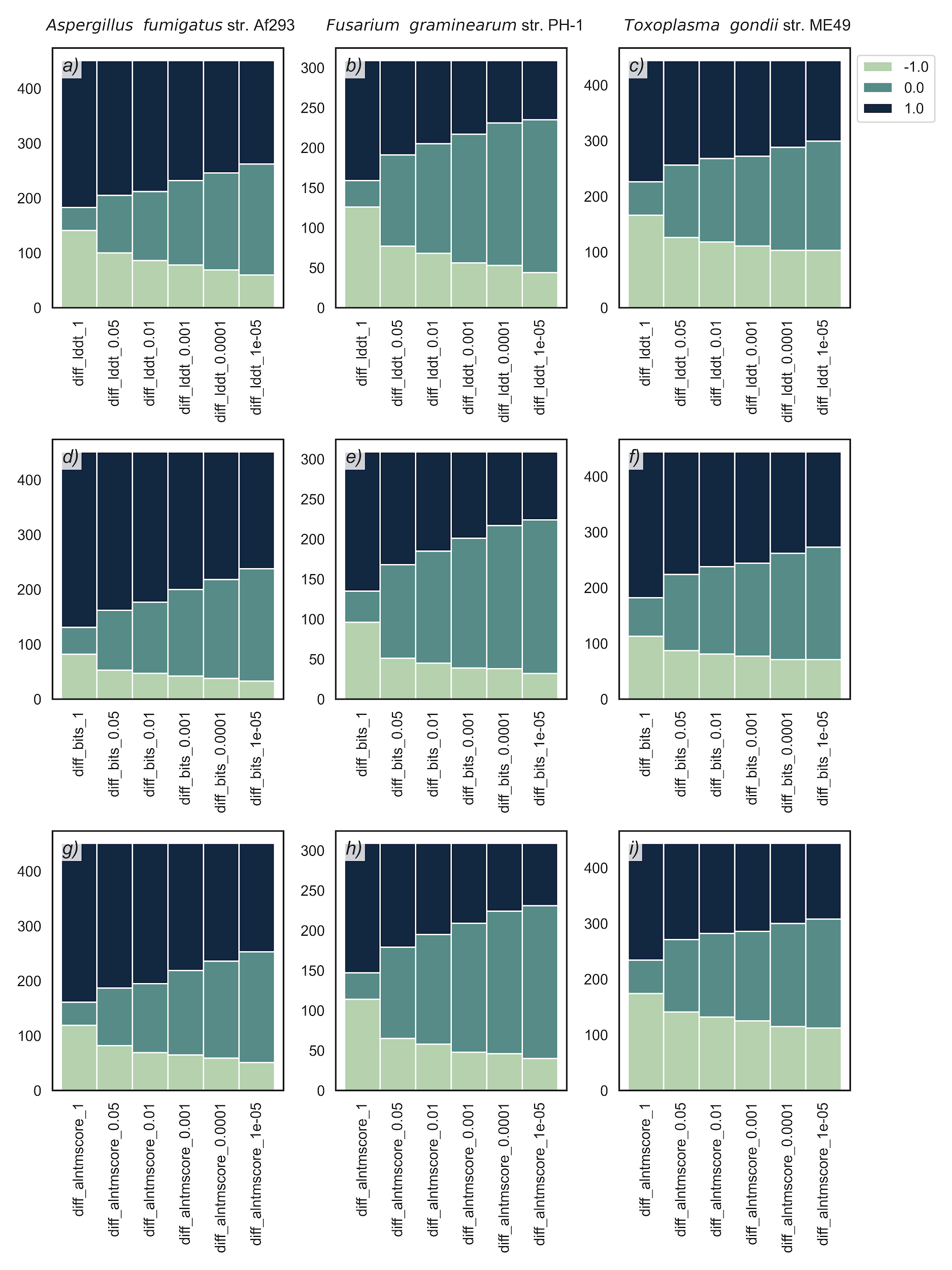
*

***Supplementary figure 9. win/loss stacked bars for Foldseek scores masked by E-value threshold.*** *Foldseek scores were masked based on whether the E-value was significant. For instance, for an E-value threshold of 0.05, all proteins with E-values greater than 0.05 would be set to zero. Wins and losses were calculated from the differences in these new masked values and scored.* *A win (+1) shows where a new model scores higher than the old model. A draw (0) is where models score the same. A loss (-1) is where the new model scores less than the old.*


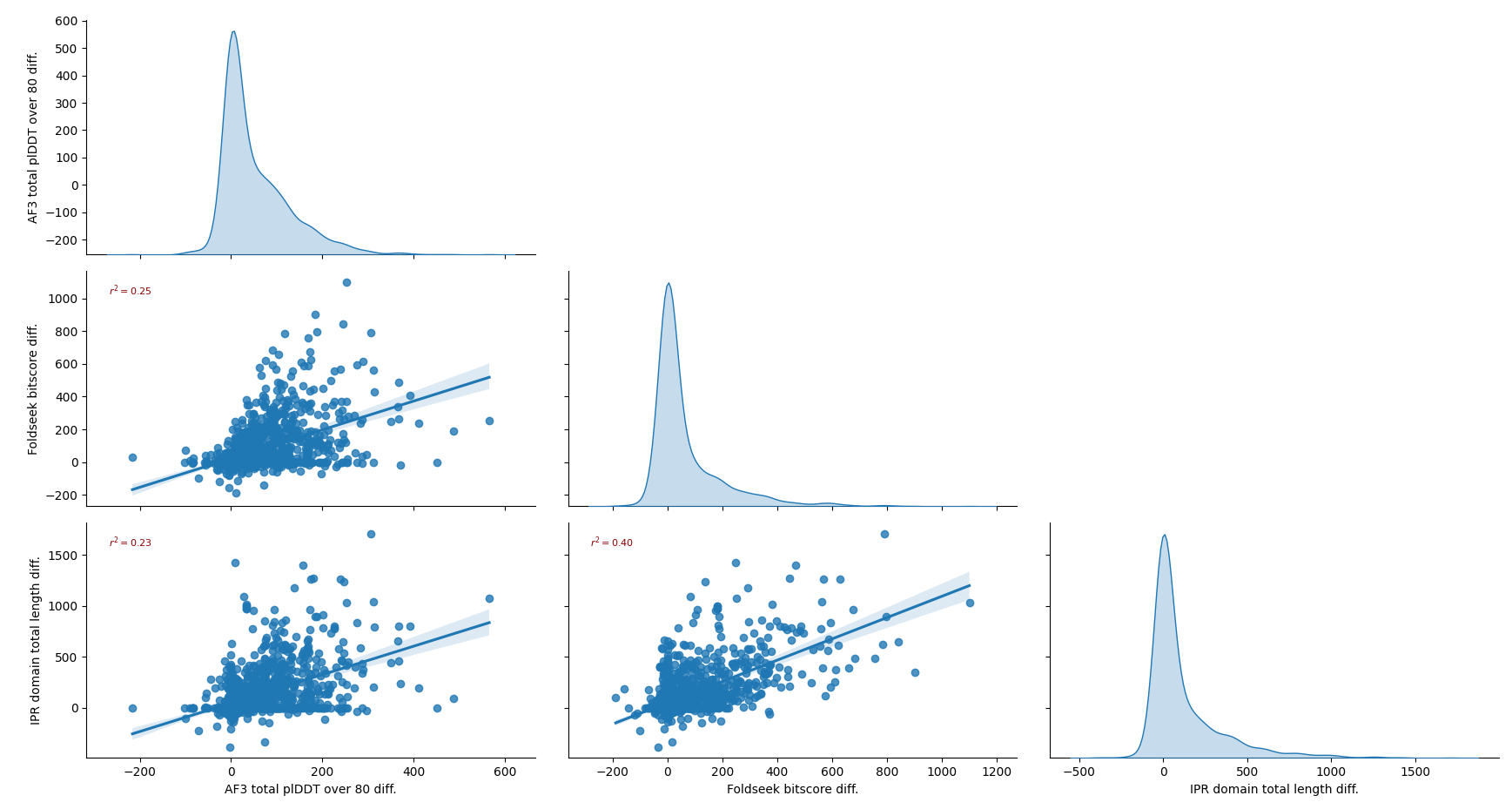


***Supplementary figure 10.*** *The correlation between the three best scores (pLDDT > 80, Bitscore, IPR total length). Scores across all three species for new models are shown.*

***Supplementary figure 11a. A clustered heatmap of the MaxAbs transformed difference values for* Aspergillus fumigatus str. Af293*.*** *In all cases pink represents a positive change from old to new models, blue represents a negative change. Scores where a smaller value is better (e.g. PAE) have been flipped to match this by -1 multiplication .*
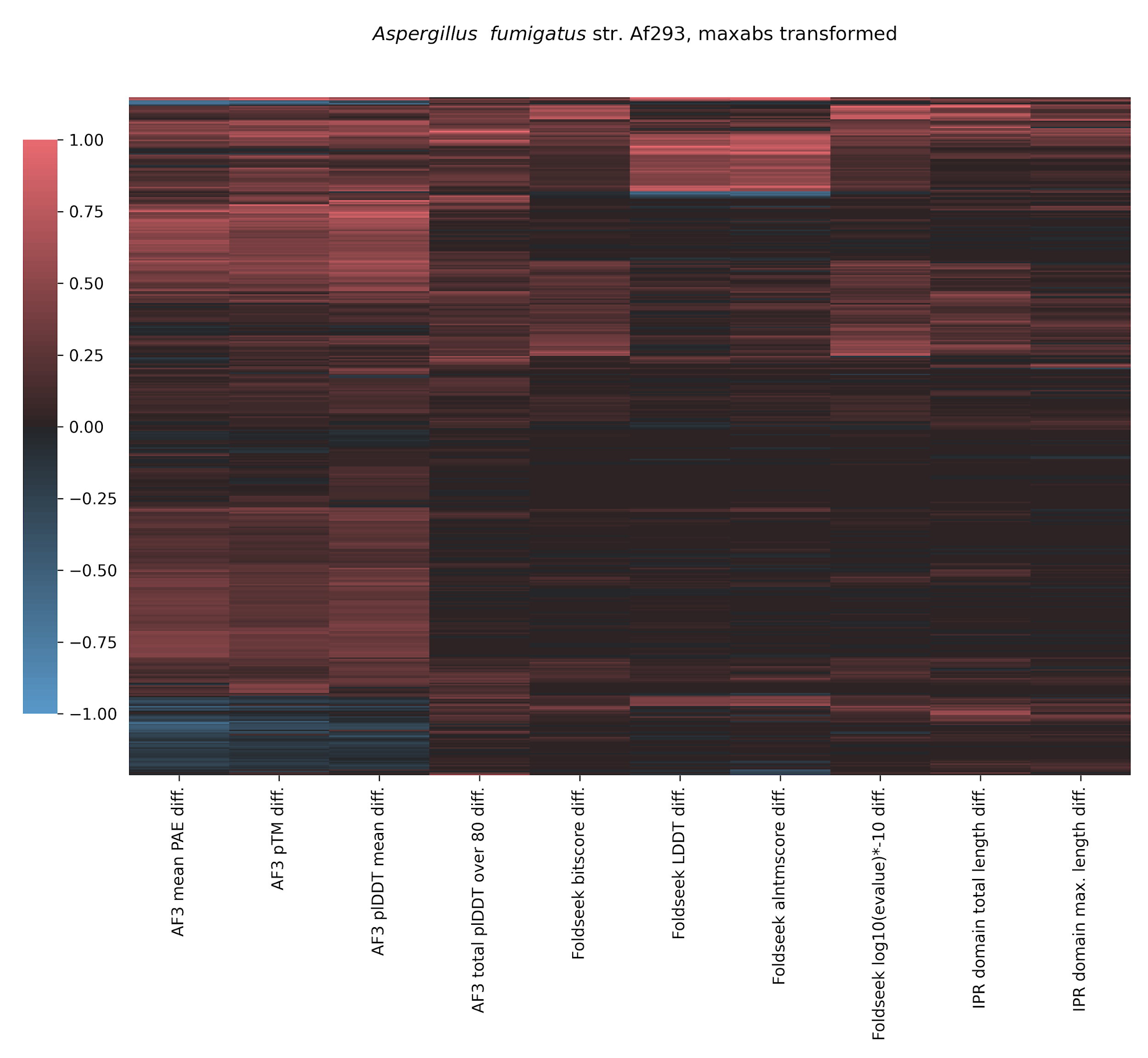


***Supplementary figure 11b. A clustered heatmap of the MaxAbs transformed difference values for* Fusarium graminearum str. PH-1.** *In all cases pink represents a positive change from old to new models, blue represents a negative change. Scores where a smaller value is better (e.g. PAE) have been flipped to match this by -1 multiplication .*
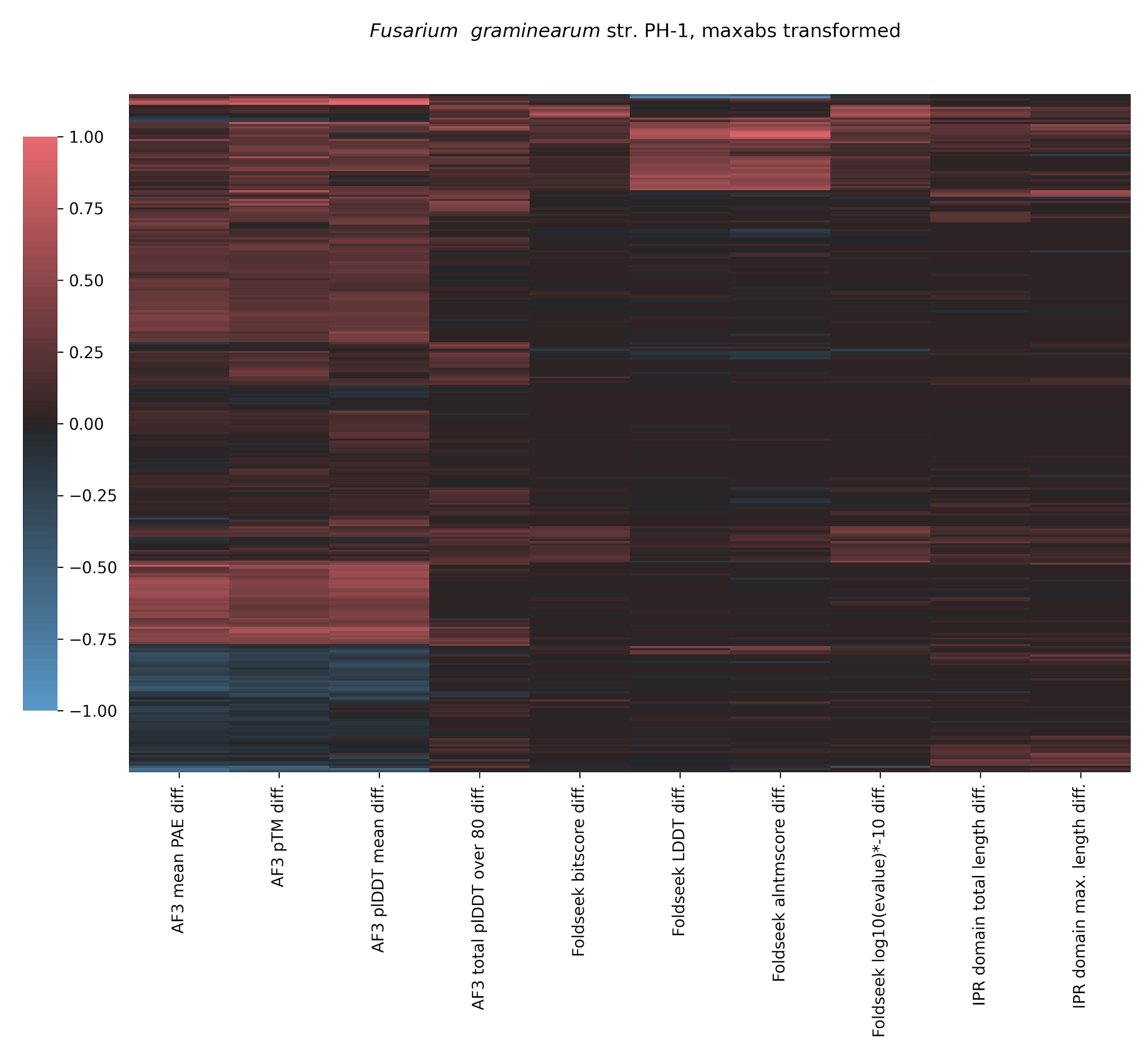


***Supplementary figure 11c. A clustered heatmap of the MaxAbs transformed difference values for* Toxoplasma gondii str. ME49*.*** *In all cases pink represents a positive change from old to new models, blue represents a negative change. Scores where a smaller value is better (e.g. PAE) have been flipped to match this by -1 multiplication.*
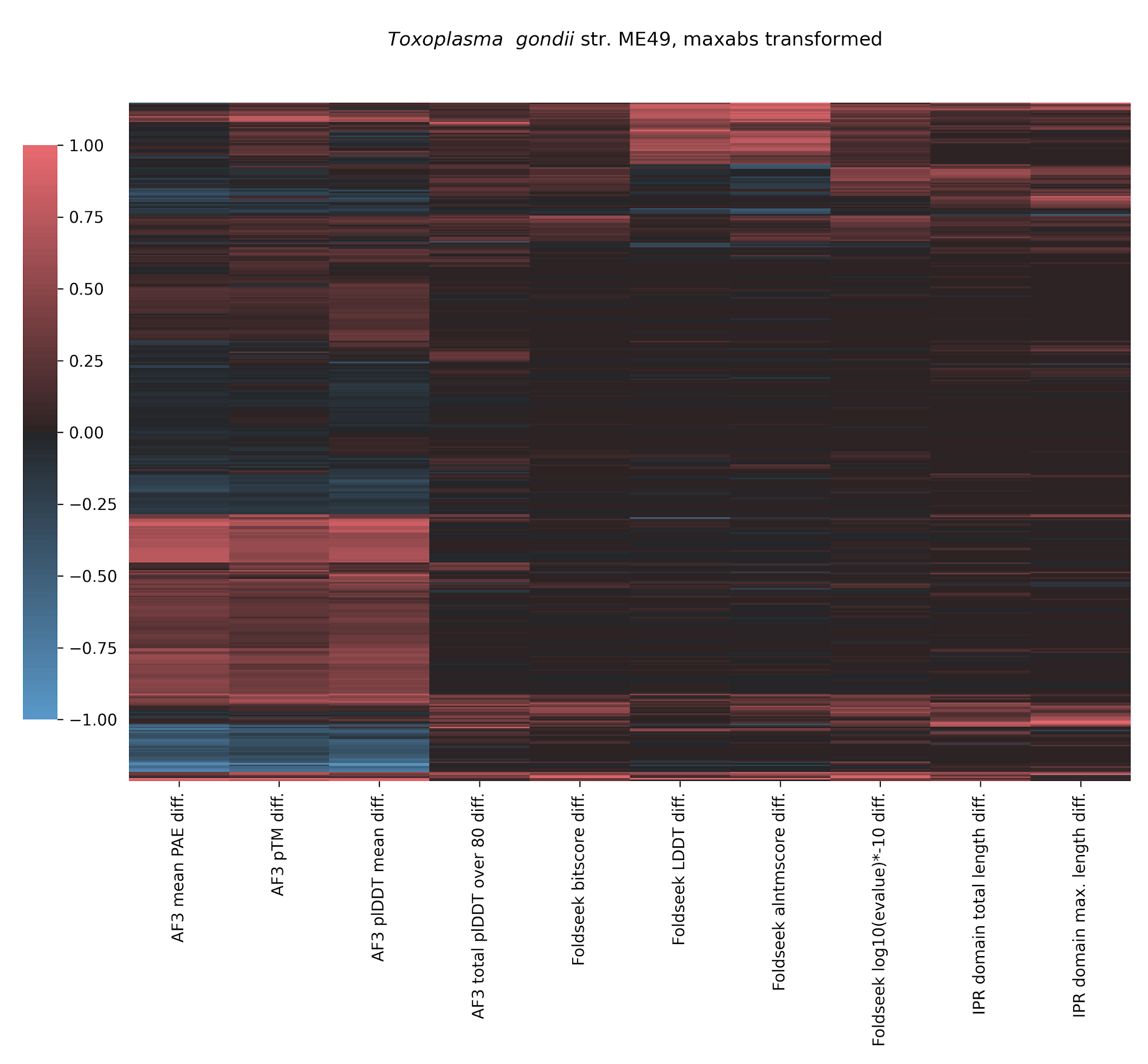


*
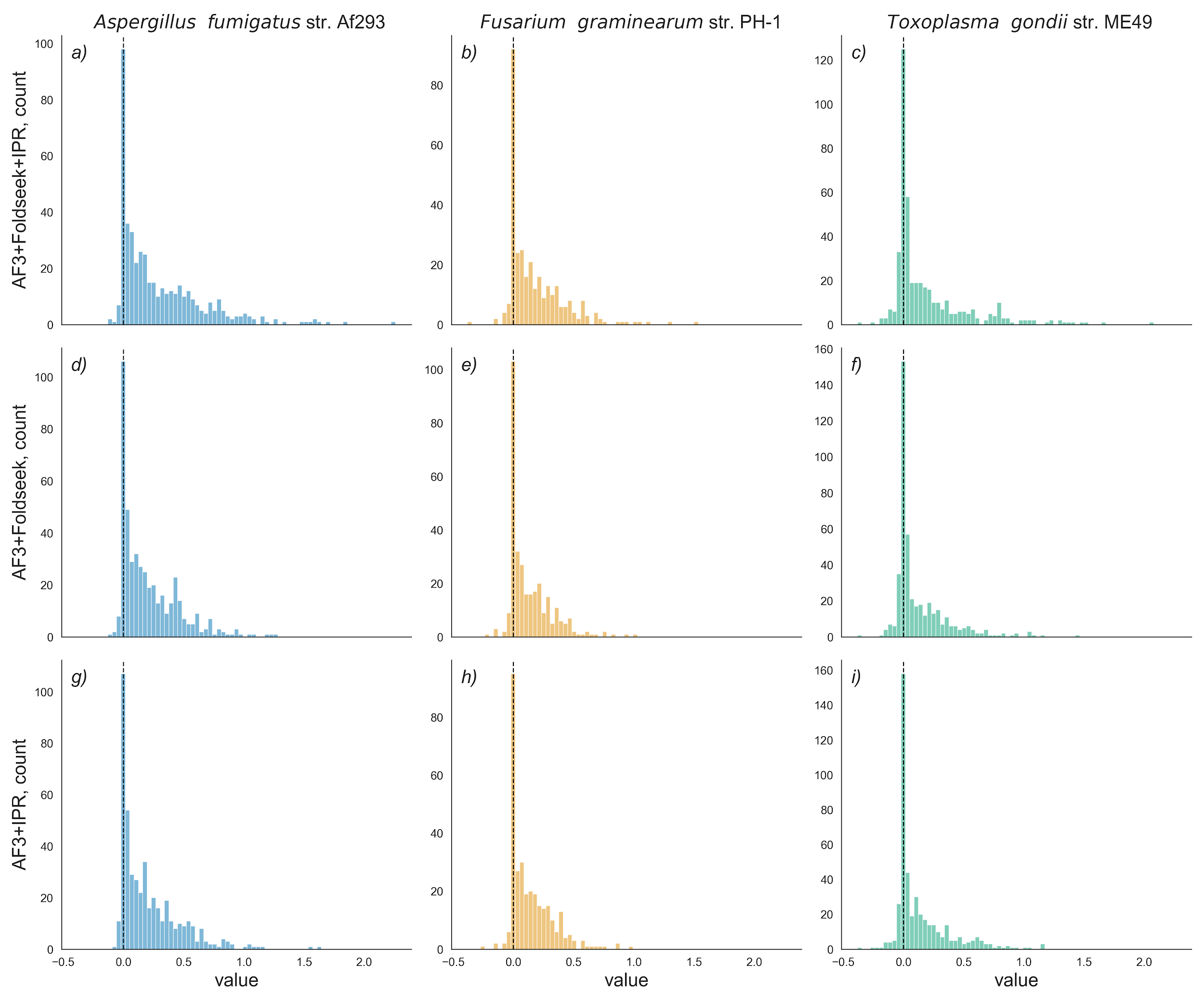
*

***Supplementary figure 12. Distribution of summed MaxAbs transformed difference scores.*** *Each row displays a different combination of the three best scores (AF3 = pLDDT >80, IPR = IPR total length, Foldseek = Bitscore on PDB search). Each column displays one species. The black dotted line indicates zero.*

*
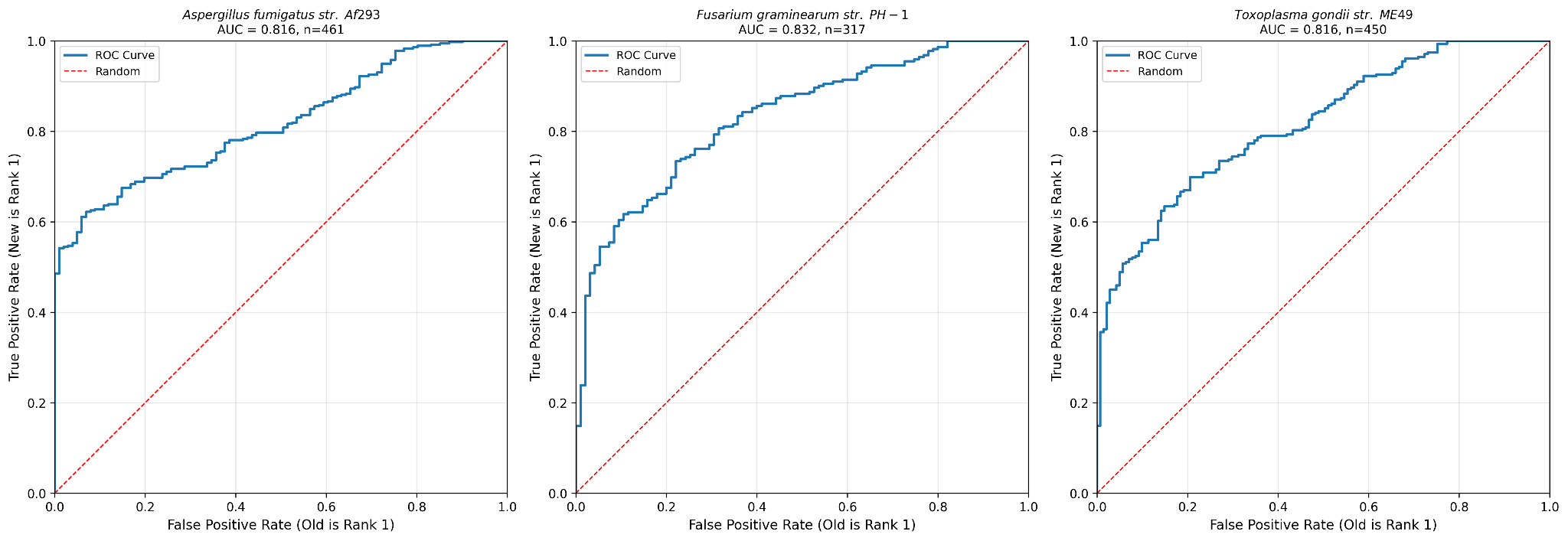
*

***Supplementary figure 13. ROC curves calculated on the basis of ranking each gene model pair (first or second, for new versus old) based on the sum of maximum absolute scaled values of pLDDT residue count (>80 threshold), FoldSeek bitscore and InterPro total domain length.*** Each score pair was then further ranked by the delta in max absolute score between the first and second ranked model, to give the ROC plot and AUC value, counting a true positive if the new model is rank 1, and a false positive if the old model is rank 1.
